# Synthesis of Radioiodinated Compounds. Classical Approaches and Achievements of Recent Years

**DOI:** 10.3390/ijms232213789

**Published:** 2022-11-09

**Authors:** Stanislav A. Petrov, Mekhman S. Yusubov, Elena K. Beloglazkina, Valentine G. Nenajdenko

**Affiliations:** 1 Department of Chemistry, Lomonosov Moscow State University, Leninskie Gory, 1-3, 119991 Moscow, Russia; 2 Research School of Chemistry and Applied Biomedical Sciences, The Tomsk Polytechnic University, 634050 Tomsk, Russia

**Keywords:** iodine radioisotopes, radiopharmaceuticals, synthesis

## Abstract

This review demonstrates the progress in the synthesis of radioiodinated compounds over the past decade. The possibilities and limitations of radiopharmaceuticals with different iodine isotopes, as well as the synthesis of low and high molecular weight compounds containing radioiodine, are discussed. An analysis of synthesis strategies, substrate frameworks, isolation methods, and metabolic stability, and the possibility of industrial production of radioiodinated organic derivatives which can find applications in the synthesis of drugs and diagnostics are presented.

## 1. Introduction

The development of radiolabeled pharmaceuticals is an actively growing area of chemistry and biomedicine. Currently, radiopharmaceuticals are applied in clinical practice as diagnostic and/or therapeutic agents. They are used for noninvasive visualization of the anatomical and physiological manifestations of various diseases (primarily oncological pathologies, as well as neurological, cardiovascular, gastrointestinal, and endocrine diseases) [1]. The use of radiolabeled compounds for diagnostics is determined by the simplicity of their detection and the possibility of determining very small amounts. As therapeutic agents, radiopharmaceuticals can be used to destroy cancer cells, leading to their death [1,2].

Radioactive halogen-containing compounds continue to play an important role in nuclear medicine because the chemical properties and nuclear decay characteristics of some radioactive halogen isotopes are ideal for use in various applications of radiopharmacy. To date, a wide range of halogen-containing radiopharmaceuticals with γ- and β-emitting radionuclides has been developed and introduced into clinical practice. Some of these radiopharmaceuticals are at various stages of clinical and preclinical trials, among which radioisotopes of iodine play an important role.

Iodine radioisotopes are applicable for labeling both low-molecular-weight compounds and peptides and nucleic acids [3,4]. Despite the widespread medical use of the fluorine-18 isotope in positron emission tomography (PET), interest in the radionuclides of iodine-125 and iodine-131 is also very high [5].

One of the advantages of radiopharmaceutical iodine isotopes is their optimal half-life. It is not as short as for other radioisotopes commonly used for diagnostics and imaging (e.g., ^11^C and ^18^F). As a result, synthetic methods of radioiodination are more diverse [4]. The longer half-life also means that radioactive iodine preparations can be used in medical institutions not equipped with a cyclotron for isotope production. The increased lipophilicity of iodine-containing compounds may also improve their pharmacological and pharmacokinetic properties [6,7,8]. Radioactive iodine itself is capable of accumulating in the thyroid gland, stomach, and salivary glands [9]; for delivery to other organs, radioactive iodine must be combined with molecules that bind to target tissues.

Comparing radioactive iodine with other halogen radioisotopes (^18^F, ^75^Br, and ^76^Br), we note the following findings. The advantage of ^18^F-containing drugs is their high percentage of positron emission (97%), as well as their relatively low energy (the low positron energy that leads to the path of the positron to annihilation with an electron is small, which allows obtaining a spatial image with a higher resolution). The disadvantage of ^18^F is its relatively short half-life (110 min); therefore, the ^123^I isotope with a longer half-life (13.2 h), despite it providing images with lower resolution, allows a wider range of radiosynthesis reactions and imaging studies.

Bromine isotopes (^75^Br and ^76^Br) are also positron-emitters and can be used for imaging. However, the production of these radionuclides is not trivial (e.g., isolation using dry distillation may be required [10]), and their nuclear properties are not optimal. For example, the ^75^Br isotope has a half-life of 97 min; a high energy of emitted positrons. In addition, it is a high-energy γ-emitter, which leads to poor imaging resolution [10].

During the last decade, some reviews have been published on synthetic methods of radioiodination [4], methods of radiohalogenation of organic compounds and their use for visualization [3], and design of radioactive iodine-containing drugs [8]. The structural features of these radiopharmaceuticals affect metabolic stability and deiodination in vivo [9]. However, a critical analysis of the methods of radioiodination of small organic molecules and macromolecules is desirable [8]. These earlier reviews have been focused on the use of radioactive isotopes of halogens in diagnostics and therapy [10], using small organic molecules [11]. However, currently, no reviews in the literature can be found that summarize the latest data on methods for the synthesis of radioiodinated derivatives with an assessment of their limitations and suitability for practical biomedical use.

A feature of the proposed review is a detailed analysis of synthesis strategies, substrate frameworks, isolation methods, metabolic stability, and the possibility of industrial production of radioiodinated organic derivatives, which can find applications in the synthesis of drugs and diagnostics. The presented data make it possible to determine priorities and choose the most appropriate synthetic approaches in the development of potential therapeutic and diagnostic compounds containing radioactive iodine.

## 2. Iodine Isotopes Used in Radiopharmaceuticals

There are 37 known isotopes of iodine, from ^108^I to ^144^I; all of them undergo radioactive decay, except for ^127^I, which is stable. Of the radioactive isotopes of iodine, ^129^I has the longest half-life of 15.7 million years. ^129^I turns into stable 1^29^Xe as a result of β-decay [12]. Historically, the first radioactive isotope of iodine, ^128^I, was obtained by Enrico Fermi in 1934 [13]. Carl Compton and Saul Hertz of Massachusetts developed a method for the production of short-lived ^128^I in small quantities, and it was used for some time in the treatment of thyroid disorders. In 1941–1943, ^128^I and ^130^I were obtained and were used for some time to treat thyrotoxicosis and thyroid cancer [13].

At present, four iodine radioisotopes (^123^I, ^124^I, ^125^I, and ^131^I) are used in diagnostics and therapy in biomedical applications. ^123^I, ^125^I, and ^131^I isotopes are included in agents for radiotherapy [14,15], and γ-emitting ^123^I, ^125^I, and ^131^I isotopes are used as imaging agents for single photon emission computed tomography (SPECT) [16,17], as well as positron emitting. ^124^I isotope is applied in PET [18].

PET is an imaging technique that uses radionuclides that decay by emitting positrons. Labeled compounds are usually introduced into the body by intravenous injection and distributed in tissues depending on their biochemical properties. Usually, 10^13^–10^15^ labeled molecules must be introduced for the study. A radioactive atom decays with the release of a positron, which annihilates further with an electron with the release of high-energy (i.e., 511 keV) photons, detected using a PET scanner. Thus, it is possible to quantitatively map the spatial distribution of radioactively labeled tracers in a living person [19].

SPECT is a tomographic imaging method that uses a γ-emitting radioisotope and radiation detection using a γ-camera. This technique makes it possible to obtain three-dimensional images, since the signal intensity in the volume element is proportional to the amount of the radionuclide [20]. Table 1 shows the comparative characteristics of the SPECT and PET methods [21,22]. It should be taken into account that SPECT tomography equipment is available in most hospitals around the world, while PET examination is much less accessible.

Iodine radionuclide therapy is used to treat several types of cancers, such as thyroid cancer, pheochromocytomas and paragangliomas, and non-Hodgkin lymphomas [13]. Therefore, patients with a thyroid tumor usually receive a radiation dose of 1100–5550 MBq (30–150 mCi) and even higher doses, depending on the type of tumor, the stage of the disease, and the tumor location, as well as the experience of the doctor [23,24]. Nevertheless, empiric dose adjustment continues to be important in the management of patients with differentiated tumors, especially in the detection of functioning metastases.

The most important properties of iodine isotopes for use in biomedicine, the physical characteristics of their decay, and information about their production methods and medical application [8] are given in Table 2.

The ^123^I isotope is a γ-emitter with a main peak energy of 159 keV, which is close to the energy of 140 keV for ^99m^Tc (one of the most common isotopes for SPECT diagnostics). The shorter half-life (13.22 h) [12] makes it possible to use ^123^I only if rapid radioactive labeling can be carried out and the resulting compounds are rapidly absorbed and metabolized. Several ^123^I-labeled imaging pharmaceuticals have been approved for clinical use by the Food and Drug Administration (FDA): ^123^I -ioflupan to visualize the dopamine transporter [29,30] and [^123^I]NaI to evaluate the morphology and function of the thyroid gland.

The ^124^I isotope was originally obtained as an impurity in the synthesis of ^123^I [12] and was not specifically studied. However, it was noted that this radionuclide has attractive properties for use in PET imaging. The half-life of ^124^I is 4.18 days, which is sufficient for clearance and localization of ^124^I-labeled monoclonal antibodies. The 23% β^+^-decay of this isotope, with the maximum and average energies of the emitted positrons of 2.138 and 0.975 MeV, respectively, makes it possible to use it for PET imaging. For comparison, the most common PET radioactive tracer, ^18^F, with 97% β^+^-decay, has maximum and average energies of emitted positrons of 0.634 and 0.250 MeV, respectively. Potentially, ^124^I can be used not only for diagnostics but also in therapy, since, in addition to the emission of positrons, ^124^I during decay emits a fairly large number of γ-quanta, most of them (63%) with an energy of 603 keV. Since ^124^I is also a β^+^-emitter and the energy of its own γ-quanta is very close to the energy of annihilation photons (511 keV) formed during the interaction of β^+^ particles with electrons, it is difficult to detect them separately from each other. Several correction methods have been proposed to eliminate this background activity, but their effectiveness is limited in the low count rates seen in clinical scanning.

The decay of the ^125^I isotope occurs mainly with the emission of X-rays with an energy of 27 keV and low-energy γ-radiation with an energy of 35.5 keV. The photon energy of this isotope is low for optimal imaging, and the half-life is long (59.4 days) [26]. Therefore, this radioisotope is commonly used in research and preclinical practice when it is necessary to label molecules or biomolecules with radioactive iodine to study their pharmacokinetics, metabolism, and biodistribution. Pharmaceuticals labeled with ^125^I, ^125^I-human serum albumin (HSA), and ^125^I-iothalamate are FDA approved for clinical use for total blood/plasma and glomerular filtration assessment.

β-Emitting isotope ^131^I (606 keV, 90%), with a half-life of 8.02 days [26], can be used for radiation therapy. The penetration depth of β-particles is from 0.6 to 2.0 mm; ^131^I is selectively taken up by thyroid tissue, and β-particles emitted by the radioisotope destroy thyroid cells with little damage to surrounding tissues. ^131^I is also a γ-emitter and can be used for SPECT imaging. In particular, ^131^I-meta-iodobenzylguanidine (^131^I-MIBG) is a radiopharmaceutical used for both imaging and treatment of certain types of neuroendocrine tumors, including neuroblastomas, paragangliomas, and pheochromocytomas. Several ^131^I-labeled compounds have been approved by the FDA, as follows:

- iobenguane ^131^I, a form of ^131^I-MIBG, for the treatment of paragangliomas and pheochromocytomas
- ^131^I-labeled HSA for the determination of total blood and plasma volume, cardiac output, cardiac and pulmonary blood volumes and circulation times; for the study of protein metabolism and borders of the heart and large vessels; and for localization of the placenta and cerebral neoplasms
- [^131^I]NaI for diagnostics and therapeutic applications

Cyclotrons, nuclear reactors, or generators are usually used for radionuclide production [31]. Cyclotrons generate magnetic fields to accelerate charged particles (protons, deuterons, and α-particles), making it possible to obtain proton-rich nuclides, which (if radioactive) usually decay owing to electron capture (EC) or positron ejection (β^+^-decay) [32]. Biomedical cyclotrons have beam energies below 20 MeV; these small cyclotrons are common in hospitals and universities. Therefore, the radionuclides that can be obtained from low-energy cyclotrons have a high potential for clinical use. Medium-energy (20–35 MeV) and high-energy (>35 MeV) cyclotrons are much less common [33].

^123^I is most often produced by direct proton bombardment of tellurium targets in a cyclotron, followed by the separation of radioactive iodine from the irradiated target. The nuclear reactions used to produce ^123^I can also be indirect, where chemically inert ^123^Xe is formed first; gaseous ^123^Xe is separated from the irradiated target, and then it decays to ^123^I. This method provides the product a higher purity than does the direct method [25].

^124^I is also typically produced using a cyclotron. However, this radioactive iodine isotope is still not available in large quantities, despite the growing demand [6].

^125^I and ^131^I radioisotopes are produced in reactors. Reactor production of radionuclides is based on the spontaneous fission of nuclei inside fuel rods, which releases neutrons capable of causing fission or neutron activation of the target material. In the latter case, the resulting nuclides are rich in neutrons and (if they are radioactive) most often decay with β-emission. Usually, thermal neutrons with a relatively low kinetic energy (E~0.025 eV) are used to obtain various radionuclides. However, for reactor production by nuclear fission, the neutron energy must be of the order of MeV [32].

^125^I is produced in a reactor and can also be obtained by the so-called (n, γ) reaction from ^124^Xe. ^125^I is commercially available as a dilute solution in sodium hydroxide of high chemical and radiochemical purity.

^131^I is the most commonly used isotope of radioactive iodine for the treatment of thyroid disorders. It is produced in a reactor and is commercially available in large quantities. ^131^I is obtained in two ways: the fission of the ^235^U isotope and the (n, γ) reaction. Since the yield of ^131^I in the chain reaction is high and radioactive iodine isotopes with a mass above 131 are short-lived, this radioisotope can be easily obtained in pure form. Irradiated uranium-235 is kept for 24 h to decay short-lived products, after which it is treated with sodium hydroxide. After filtration (to remove uranium and some fission products), the filtrate is acidified with nitric acid. Radioactive iodine is distilled off on heating and collected in a trap; the rest of the reaction mixture is subjected to further processing to separate molybdenum-99 and other fission products [6].

The (n, γ) reaction on technetium-130 leads to the formation of technetium-131m and technetium-131g. The material for irradiation target is either TeO_2_ or metallic Te. After three days, when most of the ^131^Tc has turned into ^131^I, the radioactive iodine is distilled off and collected in a trap.

^131^I is commercially available as a dilute solution in sodium hydroxide of high radiochemical purity. Sometimes a special reducing agent can be added to keep the isotope in the iodide form. However, in some cases, its use may interfere with the use of a reagent for labeling organic compounds.

In general, cyclotrons and reactors play a complementary role in the field of nuclear medicine, owing to the different nature of the radionuclides produced [34]. Radionuclide generators can also be used to produce isotopes, starting from long-lived parent radionuclides that can be chemically separated from target daughter isotopes. The parent radionuclides are applied to the resin and eluted as soon as equilibrium (temporary or long term) is reached with the daughter radionuclides, ideally resulting in the isolation of drugs of high radioisotope purity. The main advantage of using generators is an access to radionuclides independent of the cyclotron/reactor, which dramatically increases the availability of radionuclides and simplifies their clinical use.

## 3. Synthetic Methods for Incorporation of Radioactive Iodine into Molecules

The practical importance of radioactive iodine preparations requires the development of convenient preparative methods for their synthesis. However, a simple transfer of nonradioactive halogenation methods to radioactive reagents is often not possible; it requires optimization, taking into account the work with highly radioactive, short-lived isotopically labeled reagents in solutions of very low concentration. Moreover, radioiodination techniques developed for one of the isotopes of iodine may need to be modified when switching to another isotope, given the half-lives and the chemical form in which radioactive iodine is supplied. Usually, radioactive iodide is used as a starting compound in all synthesis, most often sodium iodide (Na*I) in sodium hydroxide solutions (0.01–0.1 M).

In addition to the standard indicators for the efficiency of synthetic methods (e.g., yield of target products and ease of isolation), the efficiency of the synthesis of isotopically labeled drugs is evaluated by their radiochemical yield (RCY; activity of the isolated product as a percentage of initial activity), radiochemical conversion (RCC; activity of the product before isolation, usually determined by radio-high performance liquid chromatography (HPLC), also as a percentage of initial activity) or radiochemical purity (RCP; activity of the target radioisotope relative to the total activity of all radionuclides in the sample) [35]. Another important characteristic of radioactive compounds is their molar activity (A_m_; the measured radioactivity per mole of a compound, usually expressed in GBq.µmol^−1^). High molar activity is important in imaging where it is necessary that the biological target be primarily bound with radioactive compounds, rather than with their nonradioactive counterparts. The level of molar activity required for imaging is highly dependent on the imaging method and biological target. However, in general, final compounds with molar activities of the order of GBq.µmol^−1^ are considered acceptable in the development of radioiodination methods.

Various techniques for radioactive iodination are described in the literature, depending on the nature of the organic compound being marked; however, only a few of them fully satisfied the production requirements. Ideally, the chosen iodination method should be regioselective, include minimum steps and a minimum reaction time, and provide a high RCY and high molar activity.

The general methods for the radioactive iodine incorporation into molecules were developed in the last century. They are mainly based on the methods of oxidative iodination and substitution of nonradioactive atoms for the iodine radioisotope (substitution radioiodination techniques). Early synthetic methods for radioactive iodine incorporation usually included high-temperature reactions or processes under strong oxidizing conditions, which excluded the introduction of unstable compounds into the synthesis. However, in the last two decades, a number of methods for radioactive iodination have been proposed that make it possible to circumvent these limitations and satisfy the need for new radioactive iodinated derivatives [11].

The choice of an optimal route to introduce radioactive iodine depends on the structural and steric factors in the molecules to be iodinated. Some general limitations of radiolabeling reactions should also be considered, as follows:

(1) the need to work with radioactive materials, sometimes with very high levels of radioactivity;

(2) the need to work with radionuclides with a short half-life, when reaction time, reagent concentrations, and the formation of impurities become especially important;

(3) the need to obtain compounds with high specific activity, which allows the administration of radiopharmaceuticals to patients at low doses, minimizing toxic or pharmacological side effects;

(4) the necessity for preliminary preparation and purification of iodine radioisotopes, as well as their high cost; and

(5) the preference for the simplest methods.

Requirements (1)–(3) usually imply the introduction of a short-lived radioisotope into the molecule at a late, preferably final, stage of synthesis and the need for rapid reaction and purification procedures. The total reaction and purification time should not exceed two to three times the half-life of the radionuclide.

As regards requirement (4), in most radioiodination reactions, the radioactively labeled reagent is introduced into the reaction in a minimal amount, while an excess of the substrate and other reagents is used. The reaction is carried out until the complete conversion of the reagent labeled with a radioactive isotope, which allows its maximum use.

An important difference between iodination and radioiodination reactions is their scale. Since PET and SPECT are highly sensitive techniques, and a small amount of the drug is also required for therapeutic use, radioactive compounds are administered to patients in small doses (<1^−10^ nmol) [10,36]. Because of this, and the safety concerns of working with highly radioactive materials, radiopharmaceuticals are typically prepared in micromolar amounts. As a rule, the optimization of the halogenation reaction is carried out using milligram amounts, after which reoptimization is required to adapt the technique to obtain larger amounts of radiolabeled substance. The radioiodination process is monitored by (radio-)HPLC, from which the yield and purity of the product can be assessed compared to a nonradioactive standard [37].

Requirement (5) refers to application in clinical and nuclear medicine institutions, where the simplest methods of radioiodination without the use of highly toxic precursors and reagents are preferred [3,10].

### 3.1. The Problem of Iodine-Containing Drugs’ Stability and the Optimal Position of Radioactive Iodine in a Molecule

One of the main problems in the use of radioactive iodine drugs is the low in vivo stability of the C–I bond. Elimination of the radioisotope from the drug leads to an undesirable accumulation of radioactive iodine in the thyroid gland, stomach, and salivary glands. Because of this, the signal-to-noise ratio of PET and SPECT images decreases. In therapeutic nuclear medicine, the untargeted accumulation of radioactive iodine reduces the radiation dose of target tissue and increases the radiation dose for nontarget tissues. Therefore, when developing radiopharmaceuticals, it is important to consider the metabolic transformations that they may undergo to prevent or minimize deiodination. Deiodination processes occurring in the body may be classified according to the type of enzymes that interact with radioactive iodine-containing drugs [9]:

1. Deiodinases specifically catalyze the reductive dehalogenation of an iodinated aromatic ring, most often an *ortho*-iodophenol one.

2. Cytochrome P450 (CYP450) enzymes, expressed mainly in the liver and lungs, are the main class of enzymes responsible for the oxidation of xenobiotics in vertebrates to increase their hydrophilicity. Metabolic oxidation of iodinated hydrocarbons can proceed as oxidative deiodination.

3. Nucleophilic nonspecific enzymes. Because iodide is a good leaving group in nucleophilic substitution reactions, alkyl iodides readily react with the nucleophilic SH groups of glutathione (GSH), resulting in the release of the iodide anion into the bloodstream. It is assumed that some aryl iodides are also able to react with GSH after intermediate oxidation by CYP450 [9].

An analysis of structural factors [9] made it possible to establish that molecules with radioactive iodine at *sp*^2^-hybrid carbon atoms in aryl iodides and vinyl iodides are most resistant to deiodination. These moieties are generally stable in vivo, unlike iodinated heterocycles or alkyl and alkynyl iodides. Substituents on the benzene ring of aryl iodides also affect the rate of deiodination. Summarizing the available data, the following effect of the structural features on the resistance to deiodination can be formulated [9]:

(1) Compounds with a C(*sp*^2^)–I bond are more resistant to deiodination in vivo than derivatives with a C(*sp*)–I or C(*sp*^3^)–C(*sp*)–I bonds.

(2) *meta*-Iodoarenes are generally more resistant to deiodination in vivo than their *ortho-* or *para-*isomers.

(3) Iodanilines are most often unstable to deiodination in vivo. However, the *meta*-iodonilines are more stable than the *ortho-* or *para-*isomers.

(4) Iodophenols, especially *ortho*-iodophenols, are easily deiodinated in vivo.

(5) Aryl iodides with other electron-donating substituents (e.g., alkyl and alkoxy) are more resistant to in vivo deiodination than are aryl iodides with electron-withdrawing substituents (e.g., nitro and carboxyl groups).

(6) Difluorination of aryl iodides increases their resistance to deiodination in vivo.

(7) Allyl iodides are more stable if they are attached to a nucleophilic oxygen atom rather than a nitrogen atom.

(8) Radioiodinated nitrogen- and sulfur-containing heterocycles (e.g., quinozalines, indoles, imidazoles, and thiophenes) are generally not resistant to deiodination in living systems. Iodized benzofurans, however, are resistant to deiodination in vivo.

(9) Compounds with a C(*sp*^3^)–I bond may be resistant to deiodination in vivo when (a) the other, more accessible lipophilic fragments are present in the molecule, (b) the *sp*^3^-hybrid carbon atom is included in the bulky framework fragment (e.g., cubane), and (c) iodine is part of the 2-iodoethoxy or (2-iodoethyl)amino group.

### 3.2. General Methods for Incorporation of Radioactive Iodine into a Molecule

Direct labeling of a small molecule or biomolecule with radioactive iodine usually requires an aromatic moiety, since derivatives containing iodine at the *sp*^3^- or *sp*-hybrid carbon atom are usually less resistant to deiodination (see Section 3.1). If such fragments is absent, the methods of indirect labeling using a prosthetic group is used (see Section 3.2.2) [6,34]. The prosthetic group is usually first attached to the target compound and then iodinated with Na*I in the presence of an oxidizing agent. However, if the compound to be labeled is susceptible to oxidation, it may be possible to radiolabel the prosthetic group first, followed by attachment to the target molecule.

Direct iodination methods are commonly used to introduce radioactive iodine into small molecules; macromolecules are more often radiolabeled using prosthetic groups. The problems of direct radioactive iodination of macromolecules are the following: (1) intensive deiodination of labeled molecules in vivo, and (2) possible loss of biological activity of the macromolecule under severe oxidizing conditions of direct iodination or modification of its structure. Oxidizing reagents may cause oxidation of sensitive amino acids; for example, tryptophan is converted to oxindole and methionine to the corresponding sulfoxide. Replacing these amino acids with more stable ones does not always allow maintaining the required biological effect [38]. 

#### 3.2.1. Introduction of Radioactive Iodine into Small Molecules

All isotopes of radioactive iodine are produced as iodide, usually as a solution of Na*I in NaOH (0.01–0.1 M). During radiolabeling, the iodide anion can either participate directly in the reaction as a nucleophile [10,36] or be oxidized to I_2_ or I^+^ to carry out electrophilic reactions (see below). In many early studies, radioiodination was carried out at high temperatures or in the presence of strong oxidizing agents, which limited its use for radiolabeling organic compounds with labile functional groups [11]. In recent years, new radioiodination methods under milder conditions have been developed, mainly in reactions involving transition metals [39,40,41,42,43,44].

For the synthesis of medical radiopharmaceuticals, the reactions of electrophilic aromatic substitution of a proton or a tin-containing group under the action of Na*I in the presence of an oxidizing agent (usually chloramine-T, peracids/hydrogen peroxide, or iodogen) and isotope exchange reactions in aryl iodides under the action of radioactive iodide are most often used.

Electrophilic Aromatic Substitution

**Scheme 1:**
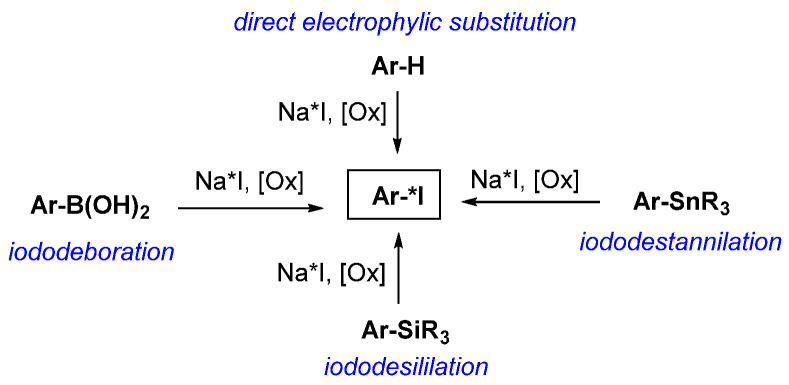
Radioiodination by electrophilic aromatic substitution reactions.

Electrophilic aromatic substitution is a popular radioiodination strategy. The starting compound may be either an arene or its tin-, boron-, or silicon-containing derivative (Scheme 1). As a radioiodine source, Na*I in neutral or basic aqueous solutions is used in the presence of an oxidizing agent. These reactions make it possible to iodinate various aromatic compounds in high RCY and RCC. Their disadvantages include a high probability of side reactions due to severe oxidizing conditions, as well as the difficulties of product purification (e.g., separation from organotin reaction products). The first problem may be solved by using a soft or solid-phase oxidizer. 

##### Substrate Choice

**Scheme 2:**
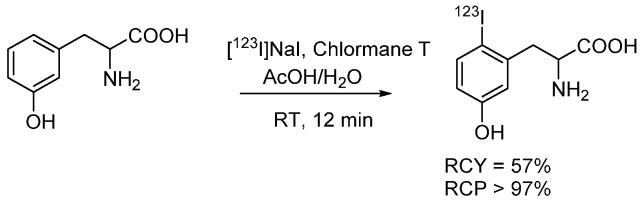
Regioselective electrophilic radioiododeprotonation [45].

*Direct electrophilic aromatic S_E_Ar substitution (iododeprotonation)* often proceeds with low regioselectivity. However, if the substrate is uniquely iodinated, the reaction may give a target iodinated product in good RCY and high molar activity [45] (Scheme 2). Nevertheless, stannylated, silylated, or borated precursors are usually prepared beforehand to overcome the regioselectivity problems, and *ipso*-substitution of these auxiliary groups is carried out [46,47,48,49].

The use of organometallic leaving groups (Scheme 1) usually suppresses the formation of substitution by-products. Moreover, owing to the electropositive nature of Sn, Si, and B, a carbon–metal bond in metallated arenes is more polar than a carbon–hydrogen one and requires less energy to break. The presence of alkyl groups attached to the metal increases the electron density on the aromatic carbon atom bound to the metal and also accelerates the electrophilic attack.

**Scheme 3:**
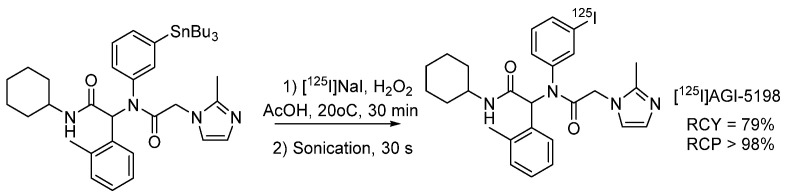
Electrophilic radioiododestannylation [51].

*Iododeostannylation* (Scheme 3) is most commonly used for electrophilic radioiodination. The starting trialkylarylstannanes are most often obtained from corresponding halogenated precursors and then radioiodinated by the action of an iodinating reagent obtained from NaI and an oxidant [45,50]. *ipso-*S_E_Ar substitution proceeds under mild conditions. The stannylation and destannylation reactions usually give high product yields, even when small amounts of starting trialkyltin (<50 mg) are used. It is important that deactivated aromatic compounds also enter these reactions, which makes it possible to obtain iodine derivatives that are stable in vivo. The main disadvantage of iododestantnylation, which complicates its use in clinical practice, is the contamination of the resulting radiopharmaceutical with traces of toxic organotin compounds. However, radioiododestannylation is currently the main method for obtaining some small molecules used as radioactive drugs, for example, [^125^I]AGI-5198 [51] or β-[^123^I]CIT [52].

In recent years, to solve the problem of purification from organotin derivatives, it has been proposed to use solid-phase extraction under the action of fluorides [53], or to carry out a reaction with stannylated derivatives in an ionic liquid followed by filtration through SiO_2_ [54]. [^125^I]SIB was isolated in 67% RCY and 100% RCC.

**Scheme 4:**
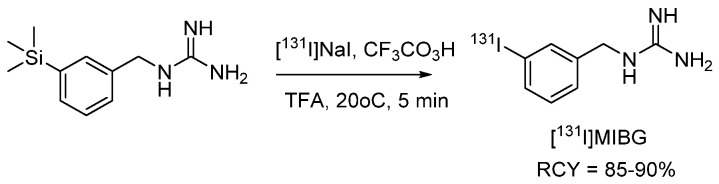
Electrophilic radioiododesilylation [56].

*Iododesilylation* (Scheme 4) generally gives the products in lower RCYs than does iododesilylation owing to the greater stability of the carbon–silicon bond. Nevertheless, iododesilylation in acidic media has been successfully used to obtain some radioiodinated derivatives, for example, [^131^I]MIBG [55] and [^123^I]iodomethomidate [56] in 85–90% RCY and 94% RCC.

**Scheme 5:**
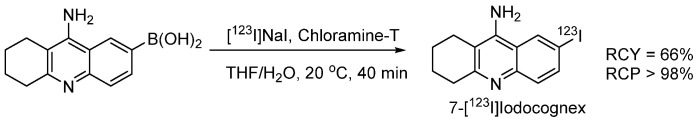
Electrophilic radioiododeboration [61].

*Iododeboration* as a method of electrophilic radioiodination (Scheme 5) has been known since the 1980s [57]. Although the C–B bond has high energy and low polarity, the empty 2*p* boron orbital facilitates electrophile attack. In addition, boron-containing compounds are readily available and nontoxic. The rate of the substitution reaction is directly proportional to the number of alkyl substituents attached to the boron atom. The small covalent radius of boron makes important the steric effect of the ligands attached to it. Often, boronic acids and their esters are also used as starting compounds [43,58,59,60,61]. Deboronation reactions proceed under mild conditions, and therefore, the substrates may contain various functional groups. However, when arenes with acceptor groups are introduced into the reaction, products are obtained in low RCYs [60,61].

##### Oxidizing Agent Choice

Electrophilic iodinating reagents are usually prepared in situ from Na*I and a strong oxidizing agent. The main task is to choose the optimal oxidizing agent that interacts with labeled sodium iodide to form an electrophilic iodine derivative (molecular iodine, iodine monochloride, etc. [3]). Sodium hypochlorite, nitrous acid, ammonium persulfate, hydrogen peroxide, iron sulfate, iodate, hypochlorite/hypochlorous acid, IBPy_2_BF_4_, penta-O-acetyl-N-chloro-N-methylglucosamine, N-chlorine derivatives of secondary amines, N-chloromorpholine, chloramine-T, iodogen, and some enzymes [3]. Reactions with peracetic acid, chloramine-T, N-chlorosuccinimide (NCS), and iodogen usually proceed in high RCYs.

**Figure 1:**
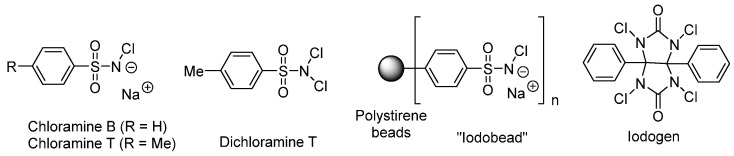
Typical oxidizing agents used in electrophilic iodination.

*Chloramine-T and its analogs.* In modern synthetic methods, sodium salts of N-chlorosulfonic acids amides, such as N-chloro-*p*-toluenesulfonic acid (chloramine-T), N-chlorobenzenesulfonic acid (chloramine-B), or N-dichloro-*p*-toluenesulfonic acid (dichloramine-T), are widely used as oxidizers of the iodide anion (Figure 1). Chloramine-T is used most often as a strong oxidizing agent in both basic and acidic media. The nature of the reaction intermediate depends on the reaction conditions, especially pH. In a neutral or weakly acid medium, the most probable oxidizing agents are ArSO_2_NHCl and HOCl [62]. Interhalogen compounds are formed in a strongly acidic medium that are undesirable for electrophilic substitution [63,64]. In weakly basic media, oxidizing agents are ArSO_2_NHCl and hypochlorite ion. Hypochlorite reacts with iodide to form HOI, which enters electrophilic substitution. In strongly basic media, IO^−^ is converted to IO_3_^−^, which is unfavorable for iodination [65]. Chloramine-T is most effective to use for iodide oxidation at pH~7 [66,67,68].

The critical factor when using chloramine-T is the temperature of the reaction mixture: the maximum RCY is achieved at temperatures above room temperature [69,70]. If the structure of the substrate allows it, the reactions are carried out at a temperature of 50 °C to 80 °C for 3–5 min. Longer reaction times can reduce the RCY due to undesirable overoxidation, leading to the products of polymerization, chlorination, and oxidation of thiol groups [14,15,71,72]. To avoid overoxidation, using high concentrations of chloramine-T is also not recommended. Chloramine-T is not suitable for protein iodination, as it causes their oxidative denaturation. Moreover, radioiodination using chloramine-T often proceeds non-regioselectivity; however, in some cases, regioselective iodination is possible using an organometallic precursor (Scheme 1) [73].

A variation on the use of chloramines is the so-called *iodobead method*. The iodobead reagent is chloramine-T immobilized on polystyrene granules [74]. Unlike free chloramine, iodine granules do not cause structural changes in proteins and can be used for their radiolabeling without loss of biological properties. Other attractive properties of iodine granules are ease of use, long storage period, minimal reaction time (in some cases only 2–15 min), a wide range of permissible pH (5.5–8.5), the possibility of carrying out reactions at room or low temperature (up to 4 °C) [74], and the ability to slowly release the oxidizing agent into the solution while maintaining its low concentration [75,76]. In addition, this oxidizing agent is easily separated from the reaction solution by filtration [77] and makes it possible to carry out reactions in organic solvents in which mono-chlorosulfonamides are insoluble, which limits their use in aqueous media [78].

*Iodogen* (1,3,4,6-tetrachloro-3a,6a-diphenylglycouryl; Figure 1) is the second most commonly used oxidizing agent in radioiodination. It makes it possible to obtain RCYs comparable to those of chloramine-T, but causing minimal oxidative damage to proteins, peptides, and cellular membranes [79,80]. Iodogen is insoluble in water; therefore, to carry out reactions in an aqueous medium, it is usually dissolved in a low-boiling-point organic solvent (dichloromethane or chloroform) and transferred to a reaction vessel, and the solvent is allowed to evaporate to form a thin layer of the oxidizing agent on the walls of the vessel [81]. It allows effective iodination of aromatic substrates containing activated aromatic groups [81]. Iodogen is a mild oxidizing agent; the probability of side oxidative reactions when using it is minimal [82], since iodogen is hydrophobic by nature and, when reactions are carried out in an aqueous medium, it reacts only at the phase boundary [83]. The concentration of iodogen plays a key role in radioiodination. The use of its high concentrations may reduce the RCY due to precipitation of the oxidizing agent from the reaction mixture [79,80,81,84]. When the reactions with the substrates resistant to oxidation are carried out in organic solvents, RCYs increase to 95–100% [85]. The pH of the reaction mixture is also important to obtain a high yield; usually, the oxidation is carried out at pH 7–8. 

**Scheme 6:**
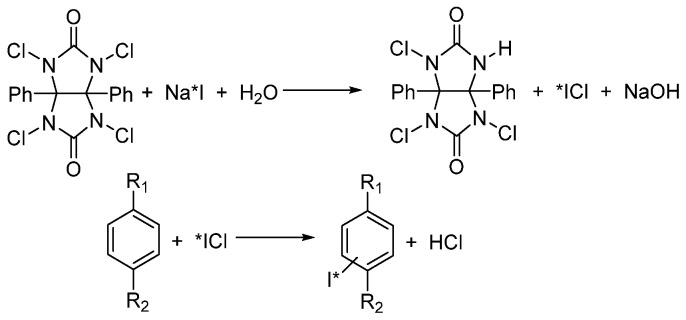
Electrophilic iodination using an iodogen.

The proposed mechanism of oxidative iodination using an iodogen is shown in Scheme 6. By the action of an iodogen, both direct electrophilic aromatic substitution of hydrogen and *ipso*-substitution of metal-containing groups are possible [8].

**Scheme 7:**
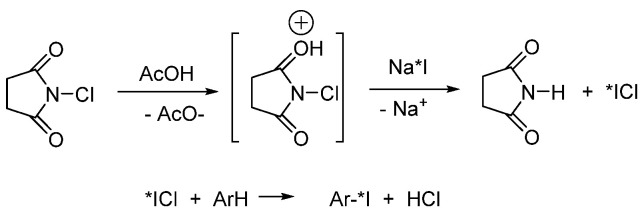
Electrophilic radioiodination using N-chlorosuccinimide.

*N-Halosuccinimides,* such as N-chloro-tetra-fluorosuccinimide, NCS, and N-bromosuccinimide, are very good oxidizing agents for radioiodination. Their reactivity may be controlled using differing catalysts, solvents, and substrates. N-Halosuccinimides are readily available and relatively stable compared to other N-Hal reagents. Aromatic compounds with donor substituents are iodinated using NCS and sodium iodide in acetic acid (Scheme 7) in a short time and in high chemical yields [86].

NCS and sodium iodide in trifluoromethanesulfonic acid can also be used for direct electrophilic radioiodination of nonactivated and strongly deactivated arenes, isoxazoles, and pyrazoles [87,88] if the reacting compounds are stable in trifluoromethanesulfonic acid. BF_3_–H_2_O can also be used instead of protic acids [88]. N-Iodosuccinimide is effective for iodination of a wide range of substrates, from phenols and anilines to nitrobenzene. It can also be used for iodination of nonbenzenoid aromatics and heteroaromatic compounds. However, N-iodosuccinimide as an oxidizing agent gives a very low RCY, and at the same time, radical side reactions are observed. NCS may also be used for protein radioiodination followed by conjugation [89] (see Section 3.2.2).

**Scheme 8:**
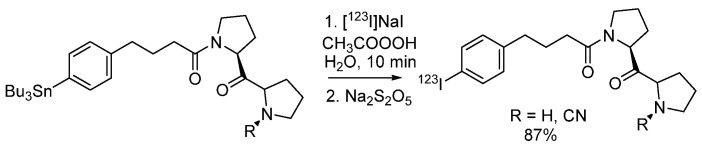
Electrophilic iodination with peracetic acid as an oxidizing agent [8].

*Peroxy acids as oxidizing agents.* Radioiodination with organic peroxy acids often proceeds quickly, under mild conditions, and with minimal formation of by-products. Aliphatic peracids are generally soluble in water and organic solvents, allowing for a wide range of reaction options. The simplest of the peracids, hydrogen peroxide, has a low molecular weight, and its reduction product is water, which makes H_2_O_2_ a convenient and environmentally friendly reagent [90]. During oxidation with a mixture of hydrogen peroxide and an organic acid, the peroxy acid is gradually formed in the solution, so the concentration of the oxidizing agent is maintained at a low level. A comparison of the radioactive labeling of organotin arene derivatives [8] with iodogen, chloramine-T, and peracetic acid revealed that the use of a mild oxidizing agent, iodogen, results in a very low RCY of the product, the use of chloramine-T leads to a significant amount of chlorination by-products, and peracetic acid gave the target compound in 99% RCY and 99% radiochemical purity (Scheme 8). Thus, peracids can be effectively used for radioiodination of some particularly sensitive substrates [8]. 

Nucleophilic aromatic substitution

**Scheme 9:**
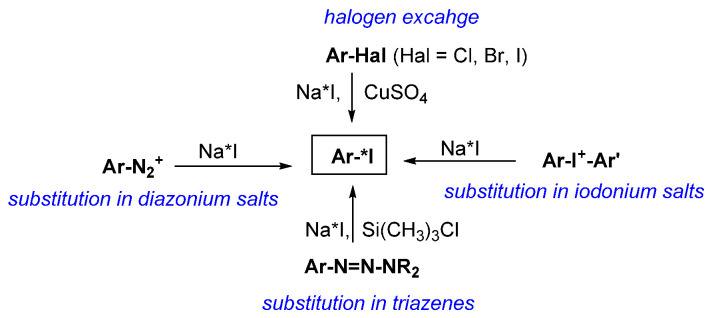
Radioiodination using nucleophilic substitution reactions.

The introduction of radioactive iodine into the target molecules by nucleophilic substitution reactions is possible if the modified substrate contains electron-withdrawing groups, good leaving groups (which usually consist of nonradioactive iodine, bromine, and chlorine), diazo groups, or iodonium groups (Scheme 9). The main problem of all methods is the separation of radioactive products from unlabeled parent compounds. To solve this problem, HPLC is often used [11,28,39,91,92,93]. In the absence of a suitable activated aromatic moiety in starting molecules, prosthetic groups may be used.

**Scheme 10:**
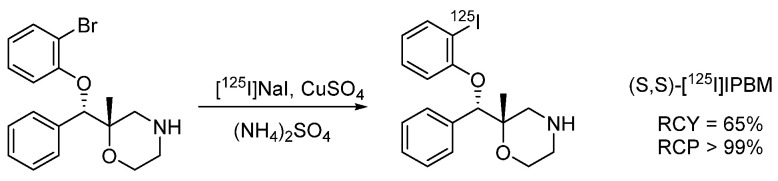
Synthesis of (*S,S*)-[^125^I]IPBM [98].

*Halogen exchange reactions* (Scheme 10) are the most common method for incorporation of iodine radionuclides into organic molecules by nucleophilic substitution reactions. To catalyze halogen exchange and increase the RCY, ammonium sulfate or copper(II) salts are added to the reaction mixture. Halogen exchange reactions are suitable to obtain products with all isotopes of radioactive iodine, although in different yields for different radioisotopes [94]. The use of nonradioactive aryl iodides for halogen exchange has the disadvantage that it becomes difficult to separate the starting material from the labeled with a radioactive isotope product. However, [^131^I]MIBG, which is usually produced by destannylation, can be also successfully obtained by the iodine–iodine exchange reaction if a high radioactive yield is not required. The advantage of iodine–iodine exchange is that it does not require the preparation of the intermediate *meta*-trialkylstannyl benzylguanidine [95,96]. When bromine or chlorine is exchanged for radioactive iodine, the debromination rate usually exceeds the dechlorination rate [8]. Reactions often proceed under harsh conditions, that is, at elevated temperatures and long reaction times, although the RCYs in these cases are usually low [97,98].

**Scheme 11:**
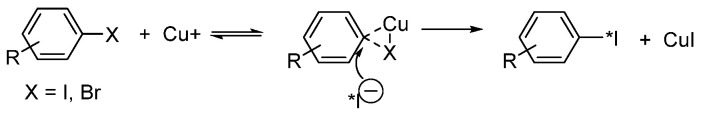
Copper(I)-catalyzed radioiodination [100].

Catalysis with copper salts improves the result of nucleophilic radioiodination [99]. It is explained by the formation of an intermediate Cu–aryl halide complex, in which the nucleophilic attack of radioactive iodine on the carbon–halogen bond is facilitated (Scheme 11) [100]. Thus, the high RCY of MIBG was obtained in such reaction at pH 1–4.4 using acids that do not have oxidizing properties [101].

**Scheme 12:**
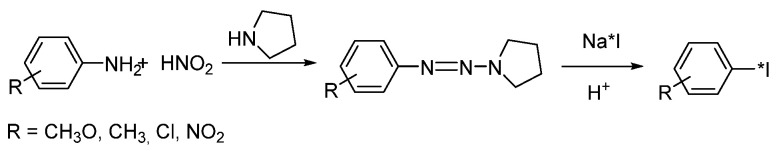
Synthesis of radioiodinated arenes with the intermediate triazene isolation [102].

*Substitution in diazo derivatives and triazenes.* Nucleophilic aromatic radioiodination reactions proceed easily for diazonium salts [102]. The problem of the starting compounds’ instability can be solved by using storage-stable triazenes as their synthetic equivalents (Scheme 12). However, the lack of general methods for the synthesis of modified precursors, the need to use them in large excess, and the possibility of side reactions make this method unpopular. Diazotization is most often carried out with sodium nitrite at a low temperature in an aqueous solution of hydrochloric or sulfuric acid, and then the resulting diazonium salt solution is treated with radioactive sodium iodide [103]. It is assumed that the reactions proceed via the S_N_1 mechanism [6]. The main disadvantage of diazotization method is the high reactivity of intermediate aryl cations or radicals. As a result, the formation of a significant amount of by-products usually takes place.

**Scheme 13:**
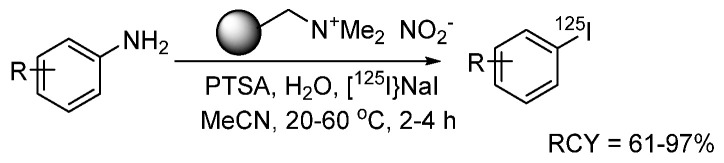
Radioiododeazotation using a polymeric reagent [104].

In 2017, Sutherland et al. [104] proposed an efficient methodology for radioactive iodination of aryl amines using a polymeric reagent to generate nitrite anions (Scheme 13). It allowed the diazotization and subsequent Sandmeyer reaction to be carried out under very mild conditions and at low concentrations of diazonium salts. The method has been tested on eight substrates with different functional groups and has been shown to be effective for the preparation of several compounds for SPECT diagnostics, including [^125^I]Iomazenil, [^125^I]CNS1261, and [^125^I]IBOX with RCYs of 47% to 75%. Triazene derivatives are less reactive than the corresponding diazonium salts, which limits their use as substrates for radiolabeling [105,106].

**Scheme 14:**
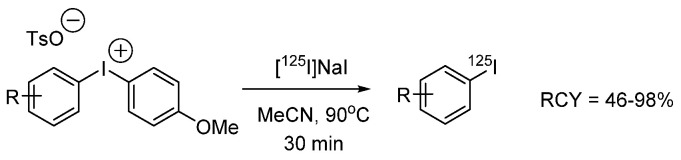
Radioiodination using iodonium salts [54].

*Iodine salts.* Gestin et al. [54] showed that the use of iodonium tosylate as a substrate allows effective radioiodination in acetonitrile at 90 °C (Scheme 14). The regioselectivity of nucleophilic substitution in unsymmetrical iodonium salts is determined by electronic and steric effects of the substituents in benzene rings [54,107,108,109]. The same authors used this methodology for radioactive labeling of activated esters, which were further used as prosthetic groups capable of acylation of biomolecules. Thus, N-[^125^I]succinimidyl-3-iodobenzoate ([^125^I]SIB) was obtained in 36–87% RCY, depending on the nature of the aryl fragment of the leaving group. In 2019, this technique was used to label two other prosthetic groups attached to biomolecules using the click or Diels–Alder reactions [107].

Reactions catalyzed by transition metals

**Scheme 15:**
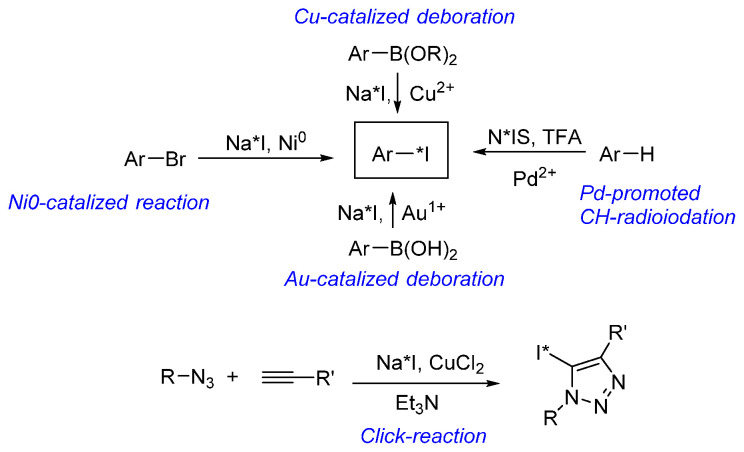
Metal-mediated radioiodination.

The problems of purification and isolation of target drugs inherent in radioiododestannylation methods have aroused interest in the development of alternative processes for radioiodination of aromatic compounds mediated by metals. The advantage of these methods is the easier separation from the metal-containing reaction products than organotin derivatives (Scheme 15).

**Scheme 16:**
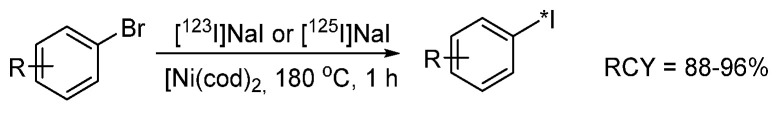
Nickel(0)-catalyzed radioiodination [39].

*Nickel(0)-catalyzed halogen exchange* was studied in detail by Sutherland in 2013 [39]. The halogen exchange reactions (Scheme 16) proceed through the oxidative insertion of Ni(0) into the C–Br bond, followed by radioiodine exchange and the formation of the Ni(II)–Br complex. Further reductive elimination of the product with a C–I* bond made it possible to obtain various aryl and heteroaryl iodides in RCYs from 88% to 96%. Radiolabeling of the SPECT tracers [^125^I]Iniparib and 5-[^123^I]A85380 occurred in RCYs of 46% and 93%, respectively; the molar activity of the resulting 5-[^123^I]A85380 was 37 GBq µmol^−1^. The resulting products were analyzed by atomic absorption spectroscopy, which showed the absence of any nickel impurities. Thus, the approach has an advantage of use over the more typical iododestantynylation procedure.

**Scheme 17:**
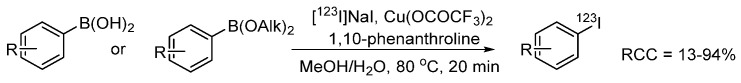
Copper(II)-catalyzed radioiodation [41].

*Copper(II)-catalyzed radioiododeboration* by the Chan–Lam–Evans mechanism was first described in 2016 by Gouverneur et al. [41] (Scheme 17). The reaction is applicable to a wide range of arenes with both electron-donating and electron-withdrawing groups, giving RCCs of 13% to 94%. Both boronic acids and their esters were introduced into the reaction; the use of the acids resulted in RCCs lower than those of esters [40,110].

**Scheme 18:**
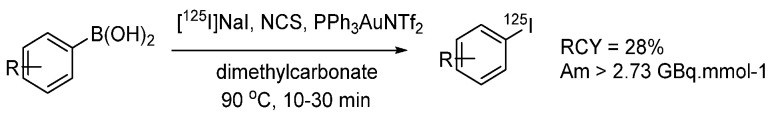
Gold(I)-catalyzed radioiodation [37].

*Gold(I)-catalyzed iododeboration* for homogeneous labeling of arylboronic acids (Scheme 18) was described in 2018 by Sutherland et al. [37]. The reaction may be carried out in air; an electrophilic iodinating reagent was generated from NCS and [^125^I]NaI. Thus, it was possible to introduce a radioactive label into electron-deficient and electron-rich substituted arenes in RCYs from 92% to 100%. Two radiopharmaceuticals, [^125^I]MIBG and the radioactive iodinated analog of Olaparib, in RCYs of 28% and 41%, respectively, have also been successfully prepared using this technique. The molar activity of [^125^I]MIBG was up to 2.73 GBq µmol^−1^.

**Scheme 19:**
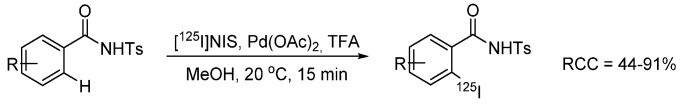
Pd-catalyzed radioiodination [42].

*Pd-catalyzed C–H radioiodination.* In 2018, Kylie’s group [42] firstly used palladium-catalyzed activation of C–H bonds in the process of radioiodination (Scheme 19). N-acylsulfonamide and palladium acetate were introduced into the reaction, and the resulting palladacycle reacted with [^125^I]NIS obtained from NCS and [^125^I]NaI. Products were obtained under mild conditions in RCCs from 44% to 91%. Other aromatic derivatives (e.g., anilides and carboxamides, N-Boc-protected anilines, urea, pyrazolylcarboxylic acids, and nitriles) also may be used in these reactions [42]. In 2019, the methodology was optimized [111] with a reduction in the palladium acetate amount while maintaining the RCC.

**Scheme 20:**
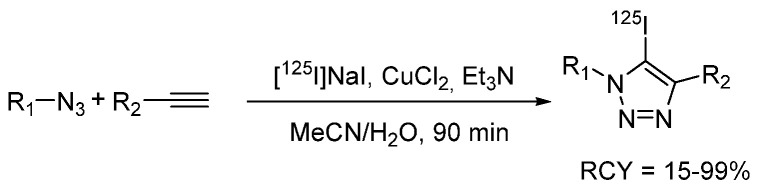
*One-pot* radioiodination according to Jan and Arstad [93].

*Radioiodination using click chemistry.* In 2013, Jan and Arstad [93] proposed a method for the one-pot radioiodination of 1,2,3-triazoles using the copper(I)-catalyzed click reaction of azide and alkyne in the presence of radioactive NaI (Scheme 20). The method turned out to be simple, versatile, and effective; preliminary studies have also shown that 5-[^125^I]iodine-1,2,3-triazoles are resistant to deiodination in vivo. Given their metabolic stability, 5-iodo-1,2,3-triazoles are promising candidates as radioactive iodinated pharmaceuticals for biomedical imaging and therapeutic applications [93].

**Scheme 21:**
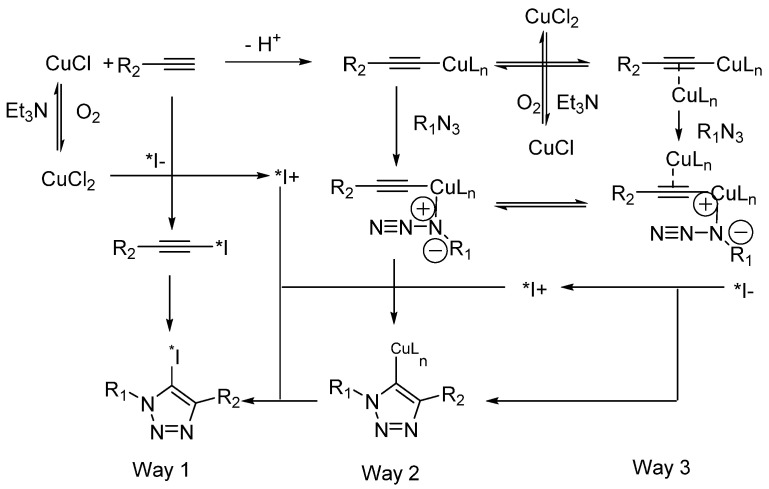
Mechanisms for the formation of 5-^125^I-1,2,3-triazoles in click reactions [93,112].

To date, various radioactive iodinated compounds have been obtained using copper(I)-catalyzed click chemistry [93,112]. Three possible mechanisms have been proposed to explain the ongoing reaction (Scheme 21), but none of them fully explain the available experimental data.

#### 3.2.2. Radioiodination of Macromolecules Using Prosthetic Groups

For radioiodination of proteins, in some cases, a direct oxidative iodination can be used; an alternative method of proteins radioiodination involves the use of prosthetic groups, which can immediately contain a radioactive label or have a functional group that may be easily replaced by iodine. For direct radioiodination of macromolecular compounds (peptides, proteins, antibodies, etc.), in most cases, oxidative iodination methods with the reagents described in Section 3.2.1 are used [91]. The most popular reagent is iodogen, which is a mild oxidizer and easily separated from radiolabeling products. The use of chloramine-T usually gives the products of chlorination, oxidation of the cysteine sulfur atom, and hydrolysis of the peptide bond. These reactions are discussed in more detail in the article of Wilbur et al. [91].

##### Direct Radioiodination

Unlike small organic molecules, proteins, and in some cases peptides, cannot be introduced into reactions under harsh conditions without losing their biological function. Direct radioiodination of proteins and peptides is therefore often impossible, although some amino acids (e.g., histidine) can be radioactively labeled with the iodine introduction into the tyrosine fragments, whose iodination proceeds under milder conditions and which are more stable to nonenzymatic deiodination. In addition, the RCY of radioactive iodination of tyrosine is usually higher than that of histidine. Radioiodination of tyrosine fragments is the most effective direct method for peptide labeling, as it is a nonpeptide modification that usually does not affect the pharmacophore conformation. However, if the tyrosine residue is important for the manifestation of pharmacological properties or is absent in the starting molecule, it is necessary to choose another radiolabeling method. In the absence of a tyrosine, it is possible to carry out direct iodination by replacing the phenylalanine presenting in the structure with tyrosine or by adding tyrosine to regions of the peptide sequence that are not critical for biological activity [28]. Direct radioactive iodination of tyrosine-containing compounds is usually carried out in phosphate buffer at room temperature for 5–10 min at a pH of approximately 7.0–7.5, using oxidizing agents such as chloramine-T or iodogen. The reaction is quenched with a reducing agent, such as sodium bisulfite, and the labeled peptides are usually purified by reversed-phase HPLC.

The main disadvantage of direct radioactive iodination is the rapid deiodination of labeled macromolecules in vivo. This disadvantage may in some cases be minimized by replacing *L*-tyrosine with *D*-tyrosine, which is less susceptible to deiodination, provided that such a replacement does not affect the function of the peptide [28]. However, in general, the problem of deiodination instability is solved by conjugating the target macromolecules with prosthetic moiety with increased biological stability.

Radioiodination of macromolecules using prosthetic groups

Unlike small organic molecules, proteins, and in some peptides, cannot be exposed to harsh reaction conditions without loss of their biological function. Therefore, an alternative strategy for indirectly labeling them using prosthetic groups has been developed. The advantages of radioiodination using prosthetic groups are as follows: (1) macromolecules are not affected by oxidizing agents, and in general, reactions proceed under milder conditions; and (2) with a properly selected prosthetic group, a macromolecule labeled with a radioactive isotope is stable to deiodination in vivo. However, the use of such a strategy also has a number of limitations: (1) the prosthetic group must be preliminarily synthesized, which requires additional reagents and work time; (2) the total RCY and molar activity of the final labeled macromolecule are usually lower than those of the macromolecule labeled using the direct method; and (3) there is a need to control unwanted modification to parts of macromolecules that are critical for their biological function [9].

**Figure 2:**
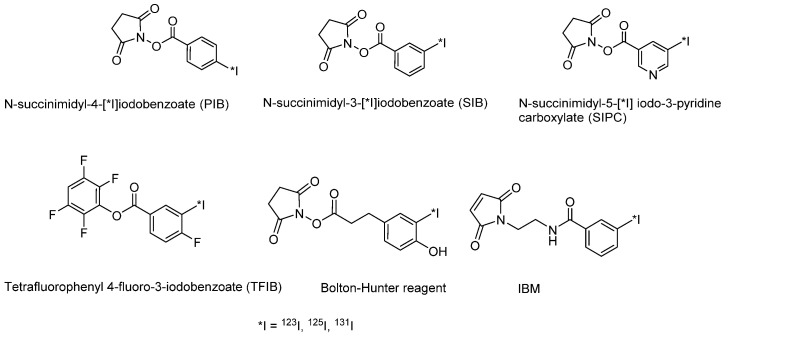

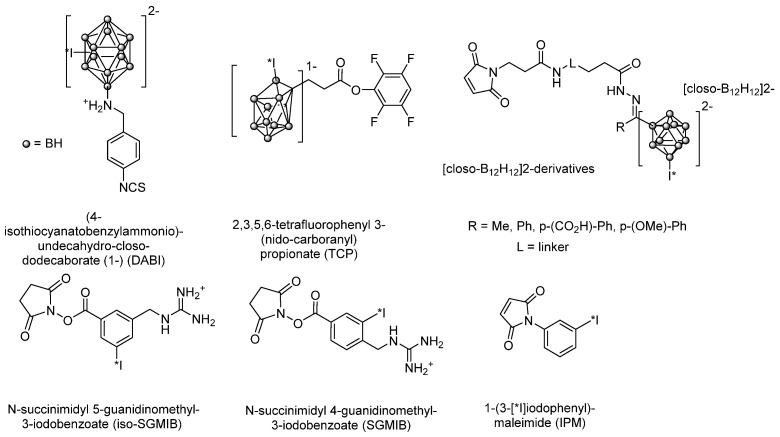
Typical prosthetic groups used in radioiodination of macromolecules.

One of the first proposed and most widely used prosthetic groups is the Bolton–Hunter reagent (N-succinimidyl-3-(4-hydroxy-5-[*I]iodophenyl)propionate) (Figure 2), which can be conjugated to the N-terminus peptide sequence or side chain amino groups (e.g., lysine). Radioiodination of the peptide is carried out in two stages: at the first stage, the activated aromatic fragment of the Bolton–Hunter reagent is radioiodinated with the necessary isotope of iodine, and then at the second stage, it is conjugated with a macromolecule to obtain the target compound [113]. The main disadvantage of all phenol-based iodinated compounds is their rapid deiodination; however, the use of Bolton–Hunter reagents slightly increases the resistance of the target macromolecule to deiodination in vivo, compared with direct labeling products [114]. 

To overcome the low stability of the Bolton–Hunter reagent and other phenolic iodinated compounds in vivo, some alternative reagents with increased stability, such as N-succinimidyl-4-[*I]iodobenzoate (PIB), N-succinimidyl-3-[*I] iodobenzoate (SIB), and N-succinimidyl-5-[*I]iodo-3-pyridinecarboxylate (SIPC), have been developed (Figure 2) [115,116,117,118]. Other maleimide derivatives, such as N-(2-aminoethyl)maleimide (IBM) or 1-(3-[*I]iodophenyl)maleimide (IPM), have also been proposed as prosthetic groups for the radioactive iodination of macromolecules [119,120].

The bimodal agent tetrafluorophenyl-4-fluoro-3-iodobenzoate (TFIB) (Figure 2) described in [121], which combines fluorinated and iodinated prosthetic groups in the structure, is suitable as an acylating agent for labeling a wide range of primary amines. [^125^I]/[^18^F]TFIB has been successfully used to label tumor-targeting peptides.

Recently, prosthetic groups based on boron derivatives, such as (4-isothiocyanatobenzylammonio)-undecahydro-closo-dodecaborate (DABI) [122], 2,3,5,6-tetrafluorophenyl-3-(nidocarboranyl)propionate (TCP) [123], and closo-decaborate(^2−^) derivatives [124], have gained popularity (Figure 2). These prosthetic groups demonstrate high resistance to deiodination in vivo and can be used for radioiodination of macromolecules. The most common prosthetic agents used for radioiodination of macromolecules by modifying the amino group are [*I]SIB, [*I]PIB, and [*I]SIPC [125].

##### Choice of Bioconjugation Reaction

**Scheme 22:**
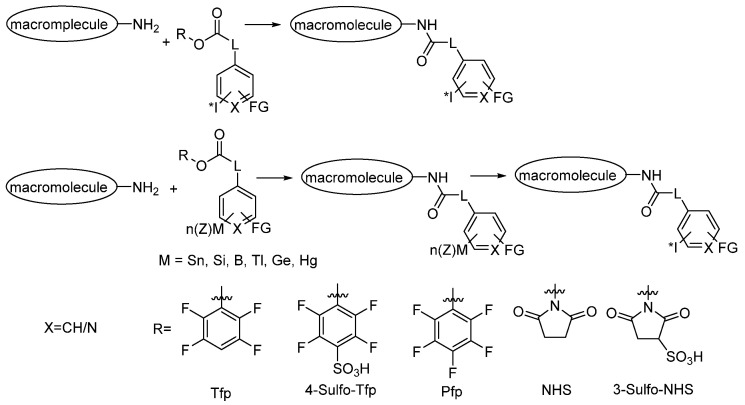
Conjugation of activated esters with NH_2_ groups of macromolecules.

**Scheme 23:**
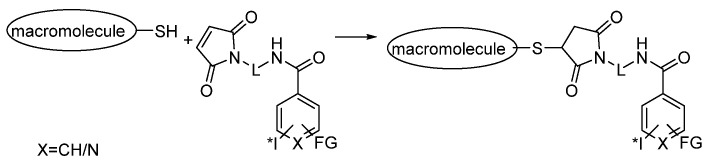
Maleimide–sulfhydryl conjugation reaction with SH groups of macromolecules.

**Scheme 24:**
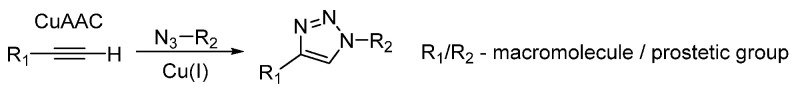
CuAAC reactions used in labeling of macromolecules.

**Scheme 25:**
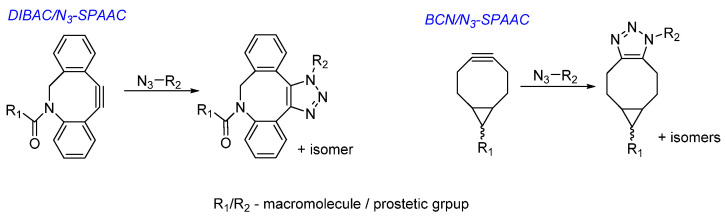
SPAAC reactions used for the labeling of macromolecules.

**Scheme 26:**
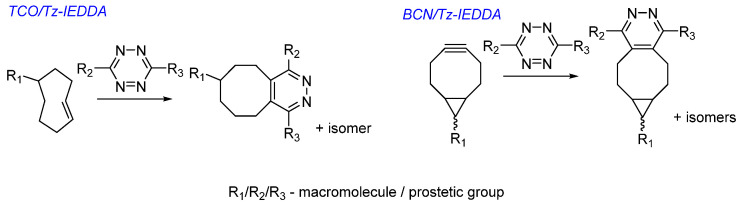
IEDDA reactions used in labeling of macromolecules.

When macromolecules are conjugated with iodine-containing prosthetic groups, amino groups (N-terminus and lysine side chain) or sulfhydryl (cysteine) groups are usually modified (Scheme 22 and Scheme 23). There is also the possibility of including nonnatural amino acids containing azido or alkyne functional groups in the peptide sequence for labeling using click reactions (Scheme 24, Scheme 25 and Scheme 26). Considering this, it is possible to use the following functional fragments for bioconjugation with macromolecule prosthetic groups: (1) NH_2_, COOH, and activated esters (NHS, Pfp, Tfp, etc.), to create an amide/peptide bond; (2) alkynes and azides, for carrying out [3+2]-cycloaddition reactions to obtain a triazole cycle (copper-catalyzed azide–alkyne cycloaddition [CuAAC] and strain-promoted azide–alkyne cycloaddition [SPAAC] reactions); (3) maleimide or SH, for maleimide–sulfhydryl reaction; (4) NH_2_ or NCS, to obtain thiourea derivatives; (5) electron-enriched dienophile or electron-deficient diene (IEDDA and Diels–Alder reaction with inverse electronic requirements); (6) SH, to obtain a disulfide derivative; (7) bromoacetyl/bromoacetamide functional groups, for the reactions with the NH– fragment of the macromolecule; and (8) imidates, for interaction with the NH_2_ groups to obtain amidines.

The formation of peptide/amide bonds between the carboxyl fragment of the prosthetic group and the amino group of the macromolecule is the most widely used approach. The prosthetic reagents used in such reactions are activated esters (NHS, N-ester of hydroxysuccinimide, Tfp, and pentafluorophenyl ether) or their water-soluble SO_3_H-substituted derivatives (Scheme 22). Conjugation is usually accomplished by reaction of the peptide with a labeled prosthetic agent in a polar solvent such as DMF or acetonitrile in the presence of a tertiary amine. If the peptide is soluble in water, the reaction can be carried out in aqueous buffer solutions with pH 8–9 or aqueous-organic reaction media with the addition of acetonitrile [121,126]. Unlike that of peptides, protein radioiodination should be carried out only in aqueous buffer solutions, ideally at pH close to the physiological range. Conjugation efficiency depends on pH, protein concentration, and temperature. Since the equilibrium content of the nonprotonated form of the amino group increases at higher pH, the efficiency of conjugation should increase with increasing pH. However, the rate of the competing reaction, namely, hydrolysis of the activated ester, also increases with increasing pH. Although Tfp esters are more stable than NHS esters at high pH, NHS esters are more commonly used. Higher yields of the conjugation reaction can be obtained at high protein concentrations, but at the expense of a decrease in the molar activity of the target product.

An example of a bioconjugation reaction through the amino group of a macromolecule via an activated ester is the preparation of a radioiodinated bovine serum albumin (BSA) conjugate using TCP as a bifunctional linker. The ^125^I-containing conjugate with this bifunctional linker was obtained in 58–75% yield and a radiochemical purity of 99.8% after purification on SephadexTM G-50. Biodistribution was studied in mice of the strain NIH and showed that the absorption of the conjugate in the thyroid gland is significantly less than that of [^125^I]-iodinated BSA [123].

Most prosthetic groups for SH fragments modification contain a maleimide function [120,127] (Figure 2 and Scheme 23). Conjugation reactions are carried out under physiological conditions and usually give relatively high RCYs. An example of introducing a prosthetic group via a maleimide–sulfhydryl reaction is the synthesis of a tumor-targeting radioiodine derivative of the F3 peptide, namely, (N-(2-{3-[^125^I]iodobenzoyl}aminoethyl)maleimide-F3Cys ([^125^I]IBMF3), which can be used as a radioligand for tumor imaging by SPECT. For this purpose, Bhojani et al. [120] synthesized a modified analog of the F3 peptide (F3Cys) containing a C-terminal cysteine residue for maleimide conjugation. [^125^I]IBMF3 was synthesized by radioiodine destannylation in an overall RCY of 73%. In vitro cellular uptake studies conducted with [^125^I]IBMF3 showed a fivefold increase in its cellular uptake after 4 h compared to controls.

Conjugation of prosthetic groups with macromolecules via click reactions (Scheme 24, Scheme 25 and Scheme 26) are carried out using three types of processes: (1) CuAAC, (2) SPAAC, and (3) Diels–Alder reaction with inverse electronic requirements (IEDDA). All these methods were used for radioactive labeling of biomolecules and in some cases were adapted for radioiodination of peptides [107,128]. Classical copper(I)-catalyzed [3+2] azide–alkyne cycloaddition (CuAAC; Scheme 24) with the formation of 1,4-disubstituted triazole is a universal click chemistry reaction applicable also in syntheses of radioactive iodine derivatives [129].

The SPAAC reactions, also known as the copper-free click reactions (Scheme 25), is a bioorthogonal reaction using a pair of reagents, cyclooctynes and azides, that react exceptionally and efficiently with each other while remaining inert to most functional groups occurring in natural compounds. SPAAC allows labeling a wide range of biomolecules without any auxiliary reagents in an aqueous or organic environment. Among known cyclooctynes, dibenzocyclooctynes (DBCOs) have a fairly high reactivity and good stability in aqueous buffers. At physiological temperature and pH ranges, the DBCO does not react with the NH_2_ and OH groups that are present in biomolecules. This method of bioconjugation has been proven effective in obtaining conjugates of various nature [130].

A bimodal ^125^I-containing cRGD-Cy5.5 conjugate was synthesized using the SPAAC reaction [131]. Radiolabeling of the stannylated precursor was carried out using [^125^I]NaI and chloramine-T to obtain ^125^I-labeled azide in a high RCY (72% ± 8%) and RCP (>99%). Using ^125^I-labeled azide, the cRGD peptide was labeled efficiently. These results clearly demonstrate that ^125^I-labeled azides may be used for efficient radioactive labeling of molecules containing the DBCO functional group.

Strained cyclooctenes and other activated alkenes and alkynes can react with tetrazines in an electronically reversed Diels–Alder reaction followed by retro-[4+2]-cycloaddition (IEDDA; Scheme 26). The driving force of this reaction is the stress relief of the eight-membered ring of *trans*-cyclooctenes. Electron-donating substituents in the dienophile and electron-withdrawing substituents in the diene accelerate IEDDA. Tetrazines bearing electron-donating groups are the suitable dienes for this reaction. IEDDA stands out from other bioorthogonal reactions with its unsurpassed kinetics and excellent orthogonality and biocompatibility, leading to a rapid growth of examples of its use in biology, imaging, and therapy [132].

A method for radioactive iodine labeling of biomolecules (cRGD and HSA) conjugated with a *trans*-cyclooctene group using the IEDDA reaction was presented [128]. The radioiodination reaction of the tetrazine structure was carried out using a stannylated precursor to obtain a ^125^I-labeled product in a high RCY (65% ± 8%) and radiochemical purity (>99%). cRGD and HAS modified with *trans*-cyclooctene were reacted with ^125^I-containing tetrazine under mild conditions to produce radiolabeled products in excellent RCYs.

Summarizing the data of this section, it should be emphasized that the applied strategy for the introduction of radioactive iodine in the case of macromolecules requires the obligatory adaptation of the general technique to each specific case, to prevent possible deiodination processes (which requires an understanding of the metabolic pathways of a radioactively labeled drug), and to preserve the biological function of the macromolecule.

### 3.3. Synthesis of Radioiodinated Derivatives: Conclusion

Thus, over the past two decades, a wide range of synthetic methods have been developed for efficient radioactive iodination of organic compounds under mild conditions. The development of these new methods and the adaptation of known iodination reactions to radiolabeling processes have made possible the rapid and efficient radioiodination of a variety of structural moieties often found in radiopharmaceuticals. Both electrophilic and nucleophilic introduction of radioactive iodine can proceed with high RCYs and specific activity of the preparations obtained. In practice, the choice of radioiodination route is determined by the electronic and steric characteristics of the compounds to be labeled. The presence of electron-donating groups in the substrate accelerates electrophilic substitution reactions. In these cases, the priority becomes the choice of the optimal oxidizing agent, which, upon reaction with labeled sodium iodide, forms electrophilic iodine derivatives that react with an aromatic substrate to form a radioactive iodinated compound. Both protons and tin-, silicon-, or boron-containing groups can undergo substitution. The disadvantage of this type of electrophilic reactions may be the undesirable oxidation of the starting or target compound. This problem can be solved by using a mild oxidizer or oxidizing agents fixed on a solid substrate. If the substrate is very sensitive to the oxidation, the prosthetic group may be radioactively labeled and then attached to the target compound. 

Electron-withdrawing groups in the substrate facilitate nucleophilic substitution reactions. Nucleophilic aromatic radioiodination can be carried out using diazonium salts, the problem of instability of which may be overcome by replacing diazonium compounds with storage-stable triazenes. However, owing to the need to synthesize a modified precursor, the use of a large excess of it, and the occurrence of numerous side reactions, the method is not very popular. 

Instead, radioactive iodination is more commonly carried out by copper-catalyzed halogen exchange in an acidic medium. The metal-catalyzed radiolabeling reactions proposed in the last decade (click reactions in the presence of copper salts and gold-, nickel-, and palladium-catalyzed processes) proceed under mild conditions and often give a high RCY. It can be expected that in the coming years some of these methods, primarily azide–alkyne cycloaddition reactions, may find application in commercial synthesis of radiopharmaceuticals.

## 4. Iodine-Containing Radiopharmaceuticals at the Stage of Clinical Trials

To date, the clinical trial database (https://www.clinicaltrials.gov/ (accessed on 1 August 2022)), funded by private and public sources around the world, contains information on more than 720 completed and ongoing phase I or II clinical trials of radioactive iodine derivatives. Both low-molecular-weight and high-molecular-weight (mainly antibodies) radioiodine compounds were proposed and investigated. 

### 4.1. Low-Molecular-Weight Iodine-Containing Radiopharmaceuticals 

#### 4.1.1. *meta*-Iodobenzylguanidine (MIBG)

*meta*-Iodobenzylguanidine (MIBG) is one of the popular radioactive iodine pharmaceuticals used with all iodine radioisotopes, such as [^123^I], [^124^I], [^125^I], or [^131^I]MIBG (also known as Azedra or iobenguane I-131 by Azedra). RFLP based on iodine-123 and 124 isotopes is used in the diagnostics at SPECT and PET. A drug containing radioactive iodine-131 was used to treat adults and children older than 12 years with certain types of malignant pheochromocytoma or paraganglioma that had spread or could not be removed surgically. It is also being studied in the treatment of other types of cancer. ^131^I-MIBG accumulates in tumor cells and emits radiation that can kill them. Thus, ^123^I-MIBG and ^131^I-MIBG, as well as ^124^I-MIBG and ^131^I-MIBG, are theranostic pairs. MIBG is currently undergoing additional clinical trials in both adults and children with relapsed or progressive neuroblastoma.

**Scheme 27:**
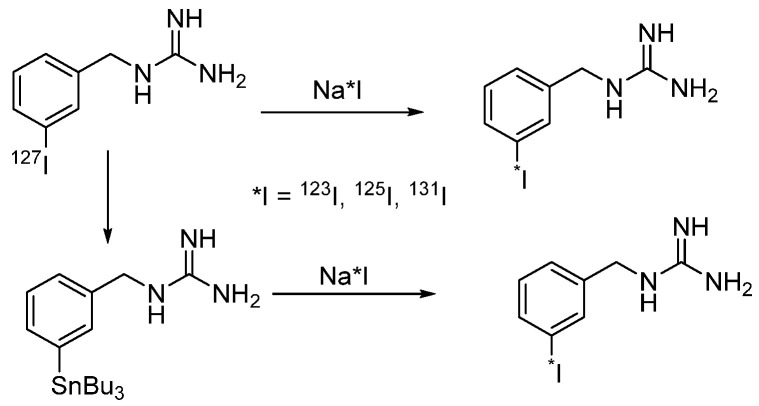
Synthesis of radiolabeled *meta*-iodobenzylguanidine (*I-MIBG) [133,134].

The main method for *I-MIBG production is isotope exchange of iodine-127 for iodine-123, iodine-124, iodine-125, and iodine-131 radioisotopes (Scheme 27). 

According to the synthetic procedure, (NH_4_)_2_SO_4_ and an aqueous solution of [^*^I]NaI are added to *meta*-iodobenzylguanidine sulfate; the resulting mixture is distilled to dryness and kept at 140–145 °C for 15–30 min. The RCY of the target product is 92–100% [133]. When the synthesis is carried out in an aqueous solution without ammonium sulfate, the reaction time is at least 72 h, and the RCY decreases to 60–80% [134]. Later, two alternative methods for the *I-MIBG synthesis were developed using the reaction of iododestantynylation by the action of [^123^/^125^/^131^I]NaI in the presence of oxidizing agents. Peroxy acids, N-chlorosuccinimide, and iodogen were used as oxidants. The organotin precursor was dissolved in acetic or trifluoroacetic acid at a concentration of 0.1–0.4 µmol/L. Then, a solution of NCS or peracid was added to the radioactive iodinating reagent, followed by an organotin derivative, and the reaction mixture was heated in an oil bath at 70 °C (a yield of 69% ± 3.1% was obtained with reaction temperature of 70 °C, while at 20 °C, the yield was only 24.9% ± 2.3%). The target product was obtained in an RCY up to 91% [55].

**Scheme 28:**
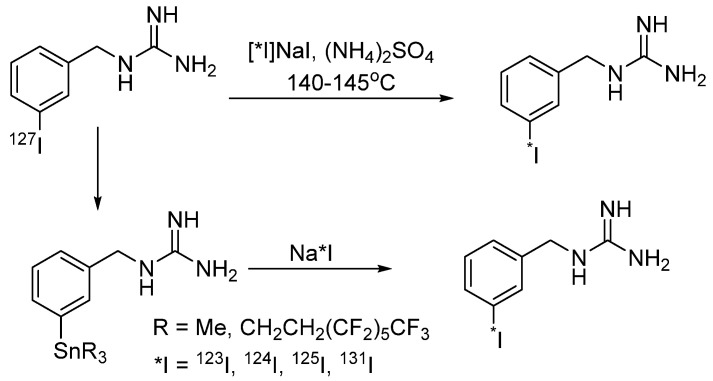
Synthesis of radiolabeled *meta*-iodobenzylguanidine ([*I]MIBG) [135].

In other synthesis (Scheme 28), 3-tris [2-perfluorohexylethylene]stannylbenzylguanidine was used as a precursor; iodogen and 5% acetic acid in methanol were added to this starting reagent, followed by sodium iodide [^125^I]NaI at pH 10. After the mixture was stirred for 3 min, the reaction was quenched by aqueous sodium metabisulphite, and after cartridge purification, the target product was obtained with an RCY of 81% [135].

**Scheme 29:**
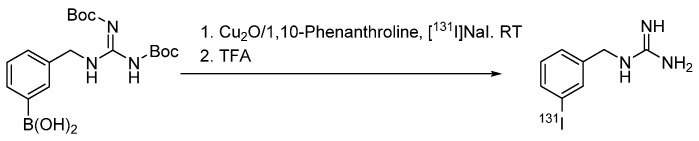
Synthesis of radiolabeled *meta*-iodobenzylguanidine ([^131^I]MIBG) [40].

Recently, a new method for the synthesis of ^131^I-MIBG was proposed based on the reaction of iododeboronation by the action of [^131^I]NaI in the presence of a copper catalyst (Scheme 29) [40]. The radioiodination procedure was quite simple: copper(I) oxide and 1,10-phenanthroline were added to the boron-containing precursor in acetonitrile, and an aqueous solution of [^131^I]NaI was added to the resulting mixture and kept for 1 h at 25 °C. The RCY after removal of the Boc protection was 98%.

#### 4.1.2. Radioiodinated Amino Acids

*para-Iodo-L-phenylalanine (IPA)*

The discovery that malignant brain tumors accumulate amino acids more actively than healthy brain cells led to the development of amino acid-based radiopharmaceuticals for the detection of brain tumors using PET and SPECT [136,137]. The iodinated amino acids *para*-^123^I-iodo-L-phenylalanine ([^123^I]IPA) and *para*-[^124^I]iodo-L-phenylalanine have been used clinically for this purpose [138,139]. Both of these phenylalanine derivatives actively cross the blood–brain barrier after intravenous administration and accumulate specifically in gliomas, presumably via the amino acid transporters L and ASC (systemic ASC amino acid transporter for neutral amino acids), which are overexpressed in malignant glioma cells [140].

A multicenter, open-label, individual phase I/II dose-finding study is currently underway to evaluate the safety, tolerability, dosing regimen, and preliminary efficacy of 4-L-[^131^I]iodophenylalanine ([^131^I]IPA) administered as single or repeated injections in patients with relapsing glioblastoma multiforme (GBM), simultaneously with second-line external radiation therapy (XRT) IPAX-1 ([^131^I]IPA and Concurrent XRT in Recurrent GBM (IPAX-1)).

**Scheme 30:**
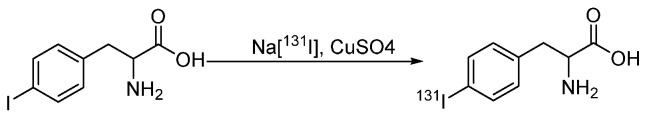
Synthesis of radiolabeled *para*-iodo-L-phenylalanine ([^131^I]IPA) [141].

[^131^I]IPA was obtained by isotope exchange for radioactive iodine in the presence of Cu(II) (Scheme 30) [141]. For this aim, a solution of [^131^I]NaI in 0.05 M NaOH and an aqueous solution of Na_2_S_2_O_5_ were evaporated to dryness by passing a stream of nitrogen through the reaction vessel at 90 °C, after which solutions of *para*-iodo-L-phenylalanine, 0.1 (NH_4_)_3_PO_4,_ L-ascorbic acid, and aqueous copper(II) sulfate were added. The mixture was heated for 60 min at 165 °C, cooled, and diluted with water. ^131^I-IPA was separated from radioactive impurities and by-products using HPLC. ^131^I-IPA was obtained with an RCY of 88% ± 10%. Synthesis of [^123^I]IPA and [^124^I]IPA was carried out according to a similar protocol.

**Scheme 31:**
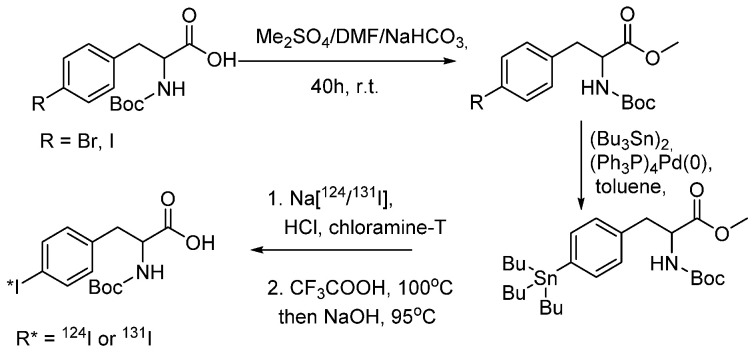
Synthesis of radiolabeled *para*-iodo-L-phenylalanine (IPA) [142].

An alternative synthesis (Scheme 31) consist of iododestannylation of the Boc-protected amino acid with [^124^/^131^I]NaI. The precursor for this synthesis is N-Boc-bromo- or iodophenylalanine. At the first stage, the halogen atom is replaced by a tributylstannyl group. Subsequently, under standard conditions, using the appropriate isotope of iodine and chloramine-T as an oxidizing agent, the tributylstannyl group is replaced [142].

*3-Iodo-α-methyl-L-tyrosine (IMT)*

**Scheme 32:**
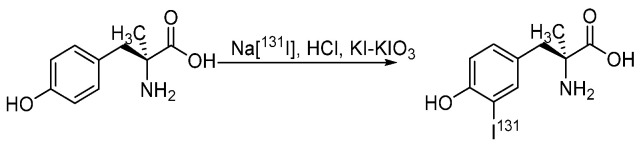
Synthesis of radiolabeled 3-Iodo-α-methyl-L-tyrosine ([^131^I]IMT) [143].

L-3-Iodo-α-methyl-L-tyrosine (IMT) is the most commonly used amino acid for SPECT studies of brain tumors. IMT demonstrates high absorption in these tumors, especially in cerebral gliomas [143]. At the same time, a number of publications are devoted to the therapeutic use of IMT in brain tumors and extracranial malignancies (Scheme 32) [143]. Since the first report of IMT radiosynthesis [144], potassium iodate has been mainly used for this purpose with some later modifications of the process [145], but chloramine-T has also been used for oxidation with comparable RCY [146]. IMT has also been reported using iodogen in a heterogeneous system [147], which is suitable for the preparation of ready-made iodination kits with cartridges for product isolation. The radiochemical synthesis yield is average (to 60–80%). An automated synthesis of IMT has recently been proposed as well [148].

#### 4.1.3. Radioiodinated Lipids

Phospholipid ethers provide a specific mechanism of action on tumor cells, since these cells contain an increased amount of lipid rafts in their cell membranes, which are believed to enhance signaling and resist apoptosis. Phospholipid drug conjugates are specifically designed to provide high affinity for lipid rafts, which upon binding, result in a transmembrane inversion with the ability to deliver the attached therapeutic drug directly to the cytosol.

*Iopofozin I-131.*

**Scheme 33:**
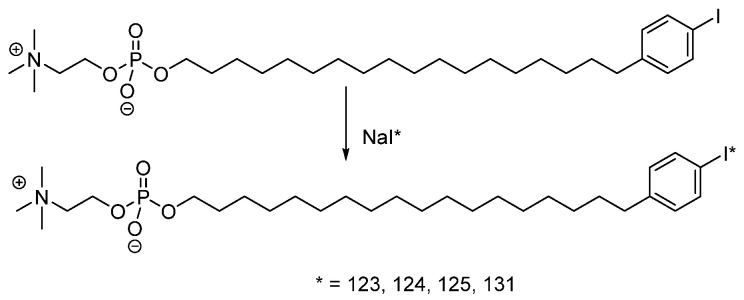
Synthesis of radioiodinated iopophosine I-131 [150].

Iopofosin I-131 (formerly known as CLR 131) is a new therapeutic agent for targeted radiation therapy of tumor cells. Iopofosin I-131 is currently in phase II clinical trials and is being studied in the groups of patients, including children and adolescents, with relapsed or refractory multiple myeloma. Iopofosin I-131 is a low-molecular-weight phospholipid conjugate [149] intended for delivery of iodine-131 directly to cancer cells while limiting exposure to healthy cells, unlike many traditional treatment options on the market. Radioiodination of phospholipid ester analogs was carried out for 7-(*para*-iodophenyl)heptylphosphocholine and 12-(*para*-iodophenyl)dodecylphosphocholine [150]. Radioiodination was carried out using the [^125^I]NaI isotope exchange reaction (Scheme 33). The radiochemical purity of the obtained labeled compounds exceeded 95% [151].

*Iodocholesterol I-131.*

Clinical studies of iodocholesterol I-131 for the diagnosis of various adrenal anomalies were completed in 2007. Prior to the injection of iodocholesterol I-131, the patient received Lugol’s solution capsules (a solution of iodine in an aqueous solution of potassium iodide) orally for two days to block ^131^I absorption in the thyroid gland. The patient was required to continue taking these capsules during the entire imaging period, which can last up to one week [152].

**Scheme 34:**
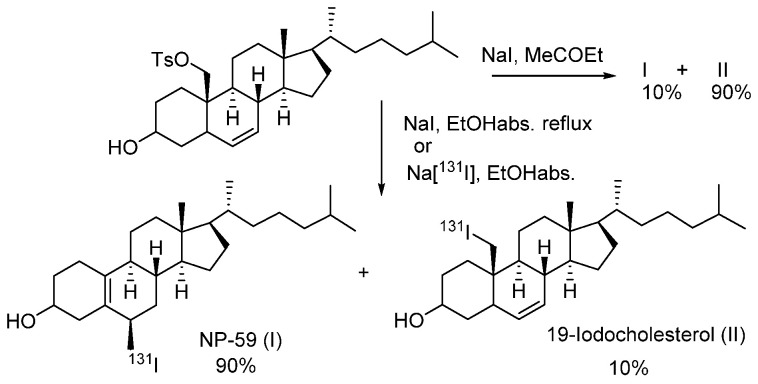
Synthesis of radiolabeled iodocholesterol I-131 [153].

During the synthesis of 19-iodocholesterol (Scheme 34), some problems had to be solved. When methyl ethyl ketone or acetone was used instead of isopropanol as a solvent, approximately 10% of an impurity was formed during the substitution of *p*-toluenesulfonate by iodide ion [153]. When absolute ethanol was used as a solvent, the amount of undesirable product increased to 90%, and the yield of the target 19-iodocholesterol was only 10%. When 19-iodocholesterol was boiled in isopropanol or absolute ethanol for 12 h, 90% of 19-iodocholesterol was converted to its isomer, 6β-iodomethyl-19-nor-cholest-5(10)-en-3−β-ol (NP-59; Scheme 34). Tissue distribution studies have shown that ^131^I-NP-59 administered intravenously to rats exhibits 24 h adrenal uptake by five times that of ^131^I-19-iodocholesterol. The ^131^I-19-iodocholesterol in the above composition at 4 °C shows slow deiodination and formation of NP-59 over time, so that the ratio of NP-59 and 19-iodocholesterol changes from 0.6 after 28 days to 3.0 after 84 days. This indicates that NP-59 is more stable than 19-iodocholesterol, and that ^131^I-19-iodocholesterol is slowly converted at 4 °C in aqueous media to NP-59.

#### 4.1.4. Radioiodinated Heterocyclic Compounds

*1-(5-Iodo-5-deoxy-β* *−D-arabinofuranosyl)-2-nitroimidazole (IAZA).*

**Scheme 35:**
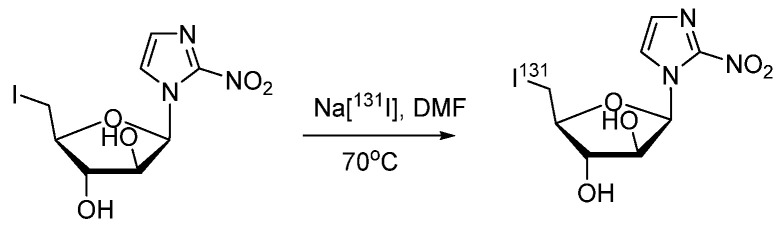
Synthesis of radiolabeled 1-(5-Iodo-5-deoxy-β−D-arabinofuranosyl)-2-nitroimidazole [155,156].

Clinical studies of [^131^I]IAZA for the detection of hypoxic tumor cells were completed in 2021, but further status has not yet been confirmed. Hypoxic cells in tumors, due to the oxygen content being lower than that of normal cells, are genetically altered and become resistant to treatment. Early studies have shown that [^131^I]IAZA imaging can help detect hypoxic cells in tumors. The experiments on laboratory animals showed the possibility of using [^131^I]IAZA as an MRI preparation [154]. The synthesis of [^131^I]IAZA and [^125^I]IAZA proceeds according to a single scheme (Scheme 35), which differs in the use of [^131^I]NaI or [^125^I]NaI. 1-(5-Iodo-5-deoxy-β−D-arabinofuranosyl)-2-nitroimidazole in dimethylformamide was added to dry Na*I, and the mixture was heated at 70 °C for 3.5 h. The cooled reaction mixture was separated by HPLC. The RCY in the exchange reaction was 75–80%; ^125^I-labeled iodide was the only radioactive contaminant detected. Radio-HPLC analysis showed chemical and radiochemical purity greater than 99% and little or no degradation of preparations within two weeks when stored at 5 °C [155,156].

*2* *β-Carbomethoxy-3* *β-(4′-((Z)-2-iodoethenyl)phenyl)nortropane (MZINT).*

**Scheme 36:**
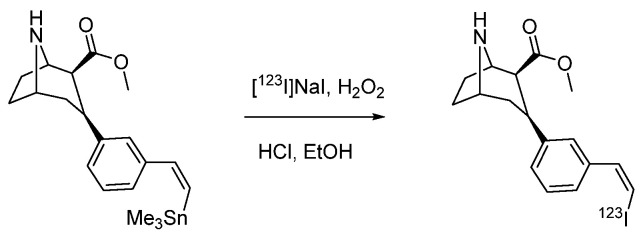
Synthesis of radiolabeled 2β-carbomethoxy-3β-(4′-((Z)-2-iodoethenyl)phenyl)nortropane [157].

One of the drugs based on the iodine-123 radionuclide for assessing biodistribution in healthy individuals and patients with Parkinson’s disease is 123-I MZINT, which completed phase I clinical trials in 2007–2009. The main aim of the studies was to evaluate 123-I MZINT as an indicator of serotonin transporters for brain SPECT in healthy individuals. The synthesis of 123-I MZINT was carried out using a ready-made kit for iodination by mixing a solution of the tin-containing substrate β-carbomethoxy-3β-(4/′-((Z)-2-(trimethylstanylethyleneyl)phenyl)nortropane in EtOH with 3% hydrogen peroxide, 0.4 N HCl, and [^123^I]NaI (Scheme 36). The reaction was carried out at room temperature for 30 min, and the reaction was quenched by adding 10% sodium bisulfite [157].

*4-(5-Iodo-2H-benzo[d][1,2,3]triazol-2-yl)-N,N-dimethylaniline (MNI-187).*

**Scheme 37:**
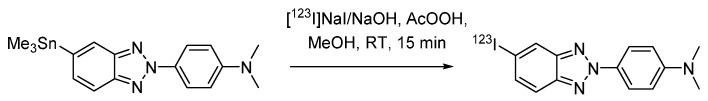
Synthesis of radiolabeled 4-(5-Iodo-2H-benzo[d][1,2,3]triazol-2-yl)-N,N-dimethylaniline [158].

To detect β-amyloid plaques in the brain of patients with Alzheimer’s disease, the drug 123-IMNI-187 was used as a marker with assessment by SPECT brain imaging. Phase I clinical studies were completed in 2007. Synthesis of 123-I MNI-187 was performed using the iododestantynylation reaction with [^123^I]NaI (Scheme 37) [158].

#### 4.1.5. para-Iodophenylpentadecanoic Acid (IPPA) and β-Methyliodophenylpentadecanoic Acid (BMIPP)

Radioiodinated fatty acids are radiopharmaceuticals of great interest because they allow tracing the metabolic pathways of fatty acids, which, in particular, are the main source of energy for a normally oxygenated myocardium. Therefore, labeled fatty acids, such as the radioactive iodine 15-(4-iodophenyl)pentadecanoic acid (*p*-IPPA), have been used in nuclear medicine protocols primarily for myocardial imaging [159]. Clinical trials of ^123^I-BMIPP completed in 2021 to test the hypothesis that renal uptake of free (i.e., nonesterified) fatty acids is increased in the event of myocardial infarction. To achieve this goal, renal uptake of fatty acids was measured in vivo for patients with myocardial infarction by SPECT/CT.

**Scheme 38:**
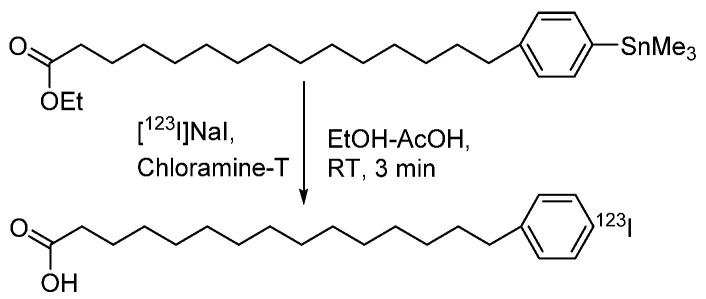
Synthesis of radioiodinated *para*-iodophenylpentadecanoic acid (IPPA) [73].

The radiosynthesis of [^123^/^131^I]IPPA and [^123^/^125^/^131^I]BMIPP is the most widely investigated. At least five methods have been developed for it: direct electrophilic iodination, iodine detailing, iododemercuration, iododestantnylation, and isotope exchange [73]. For the synthesis of ^123^/^131^I-IPPA, the most preferred method is iododestannylation of the trimethylstannylated precursor by the action of [^123^I]NaI in the presence of an aqueous solution of peracetic acid. The mixture was stirred at room temperature for 30 min and quenched with sodium thiosulfate, and then it was transferred to a 3 mL ampoule. Sodium carbonate was added, and the resulting compound was purified by passing through C-18 SepPak; the RCY was 62% [160]. Later, an express method was developed for the preparation of radioactive iodinated ^131^I-IPPA from a trimethylstannylated precursor and [^131^I]NaI using chloramine-T as an oxidizing agent in an RCY of 90% ± 1.4% and a radiochemical purity of 99% at labeling at room temperature for 3 min (Scheme 38) [73].

**Scheme 39:**
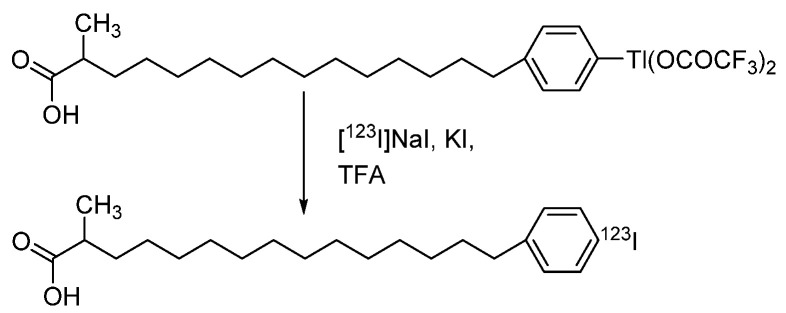
Synthesis of radioiodinated β-Methyliodophenylpentadecanoic acid (BMIPP) [161].

At the same time, for the production of ^125^I-BMIPP, the iododethaillilation of (4-(14-carboxypentadecyl)phenyl)thallium(III) 2,2,2-trifluoroacetate in trifluoroacetic acid is used as the main synthesis method (Scheme 39). After distillation of the solvent, the target product is obtained in an RCY of up to 80% [161].

#### 4.1.6. 4-(4-Fluoro-3-(4-(3-iodobenzoyl)piperazine-1-carbonyl)benzyl)phthalazin-1(2H)-one (MAPi)

**Scheme 40:**
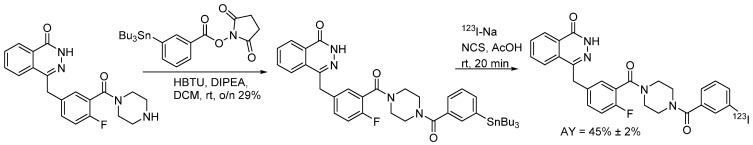
Synthesis of [^123^I]MAPi [163].

Glioblastoma multiforme is one of the deadliest forms of solid tumors with a five-year survival rate of only 5%. The use of [^123^I]MAPi makes it possible to visualize tumor progression and response to therapy, as well as calculate patient dosimetry [162]. Synthesis of [^123^I]MAPi was carried out using the iododestantnylation reaction under the action of [^123^I]NaI. Two variants of the synthesis are proposed. In the first case (Scheme 40), a radioactive label was introduced into 4-(4-fluoro-3-(4-(3-(tributylstannyl)benzoyl)piperazin-1-carbonyl)benzyl)phthalazin-1(2H)-one in a mixture of anhydrous MeCN, methanol, and acetic acid under the action of NCS, to which [^123^I]NaI in 0.1 M NaOH was added and stirred for 15 min at 25 °C. Purification by HPLC gave the target product in an RCY of 45% ± 2% [163].

**Scheme 41:**
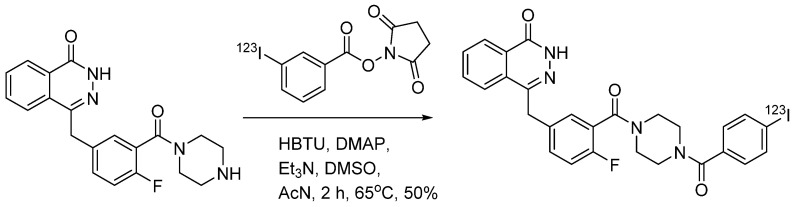
Synthesis of [^123^I]MAPi [162].

An alternative method for the ^123^I-MAPi synthesis was elaborated (Scheme 41) [162]. The reaction of iododestannylation of N-succinimidyl-3-(tributylstannyl)benzoate with [^123^I]NaI in 0.1 M NaOH was carried out. After reacting for 20 min at room temperature and distilling off the solvent, the reaction mixture was purified by HPLC. The collected purified fraction was concentrated to dryness in vacuo, dissolved in acetonitrile, and added to a solution of 4-(4-fluoro-3-(piperazin-1-carbonyl)benzyl)phthalazin-1(2H)-one in DMSO containing HBTU, DMAP, and triethylamine. The reaction mixture was stirred for 2 h at 65 °C before being purified by HPLC to give ^123^I-MAPi in an RCY of >70% and RCP > 99%.

#### 4.1.7. 4-(6-Iodimidazo [1,2-a]pyridin-2-yl)-N,N-dimethylaniline (IMPY, TZDM)

**Scheme 42:**
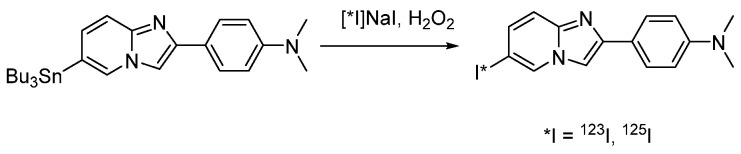
Radiolabeled 4-(6-iodimidazo [1,2-a]pyridin-2-yl)-N,N-dimethylaniline [164,165].

To estimate the effectiveness of the biomarker [^123^I]IMPY (or [^125^I]TZDM) in SPECT investigation as a drug for detecting β-amyloid in the brain of patients with Alzheimer’s disease, clinical studies were conducted in 2006–2008 [164,165]. For the synthesis of [^123^I]IIMPY, the iododestannylation reaction of N,N-dimethyl-4-(6-(tributylstannyl)imidazo [1,2-a]pyridin-2-yl)aniline with [^123^/^125^I]NaI in the presence of hydrogen peroxide as an oxidizing agent was used (Scheme 42).

#### 4.1.8. Iodobenzovesamicol (IBVM)

**Scheme 43:**
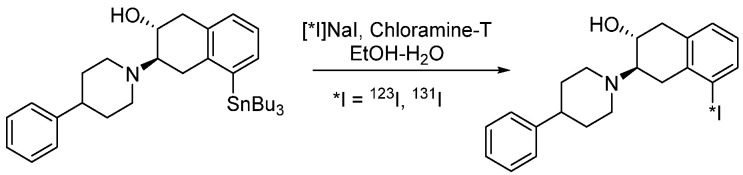
Synthesis of radiolabeled *S*-iodobenzobenzovesamicol [167].

*S*-[^123^I]Iodobenzovesamicol ([^123^I]IBVM) is a highly specific in vivo marker for cholinergic neurons in the brain. Clinical evaluation of [^123^I]IBVM as a SPECT agent for noninvasive mapping of cholinergic nerve loss in patients with dementia disorders, such as Alzheimer’s disease and Parkinson’s disease, was completed in 2011 [166]. Synthesis of 123-I IBVM (Scheme 43) was carried out in a transport vial containing [^123^I]NaI in 0.1 M NaOH, to which was added 0.2 N ethanol solution of H_2_SO_4_ and then (-)-5-(tri-n-butyltin) benzovesamicol, controlling the pH of the reaction mixture (pH 4–5). The reaction was initiated by adding a freshly prepared aqueous solution of chloramine-T. The target product was obtained in an average radiochemical purity and a chemical purity of more than 97% [167].

#### 4.1.9. 4-(2-(Bis(4-fluorophenyl)methoxy)ethyl)-1-(4-iodobenzyl)piperidine (β-CIT)

**Scheme 44:**
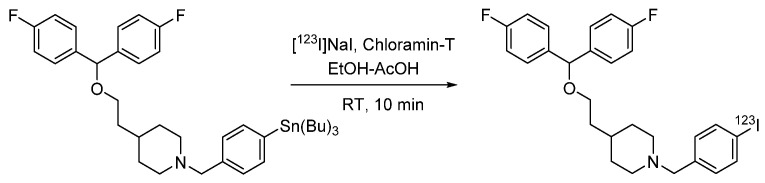
Synthesis of radiolabeled 4-(2-(bis(4-fluorophenyl)methoxy)ethyl)-1-(4-iodobenzyl)piperidine [169].

[^123^I]4-(2-(Bis(4-fluorophenyl)methoxy)ethyl)-1-(4-iodobenzyl)piperidine (β-CIT) is used for imaging assessment of dopamine transporter density in patients with early Parkinson’s disease participating in early and late levodopa therapy for Parkinson’s disease. Phase II clinical trials were completed in 2007 [168]. β-CIT was obtained by electrophilic radioiododestannylation of 4-(2-(bis(4-fluorophenyl)methoxy)ethyl)-1-(4-(tributylstanyl)benzyl)-piperidine in ethanol containing glacial acetic acid, chloramine-T in deionized H_2_O, and [^123^I]NaI in 0.05 M NaOH (Scheme 44).

#### 4.1.10. Ioflupan (FP-CIT)

**Scheme 45:**
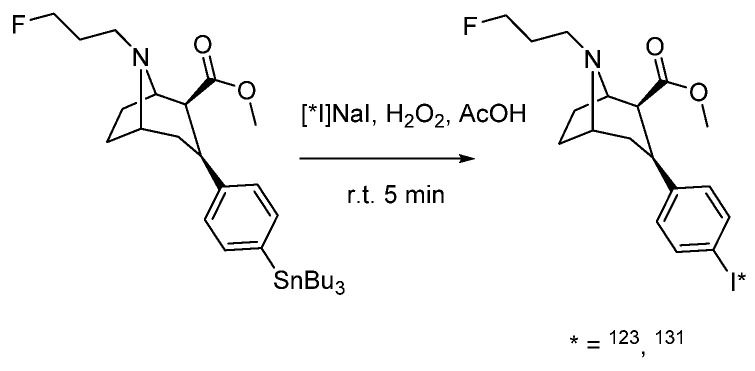
Synthesis of radiolabeled Ioflupan [171].

123-I Ioflupan underwent clinical trials in the USA in 2011 and was recommended for diagnosis in Parkinson’s disease. Studies were conducted in 2021 to evaluate the safety, biodistribution, internal dosimetry, and effective dose of DaTSCAN™ injection of ioflupane (123-I) in healthy volunteers. Ioflupan ([^123^I]FP-CIT or [^131^I]FP-CIT) is used for imaging nigrostriatal pathway and can be a valuable tool when the clinical signs are not clear enough. Parkinson’s disease and Parkinsonian syndromes are movement disorders that present with nigrostriatal degeneration with reduced levels of the dopamine transporter, and hence, reduced distribution of ^123^I-ioflupan [170]. In some cases, the iodine-123 isotope can be replaced by iodine-131 using [^131^I]NaI. Ioflupane (123-I or 131-I) is produced by iododestannylation and is usually carried out manually in a small reaction volume (Scheme 45). The optimal reaction conditions are the use of hydrogen peroxide in acetic acid with the addition of [^123^I]NaI or [^131^I]NaI to the tin-containing precursor, which leads to an almost quantitative RCY (98.0% ± 0.5% according to HPLC data) [171].

#### 4.1.11. (2R)-2-(2-Hydroxy-1-iodopropan-2-yl)-8,9-dimethoxy-1,2,12,12a-tetrahydrochromeno [3,4-b]fluoro [2,3-h] chromen-6(6aH)-one (CMICE-013)

**Scheme 46:**
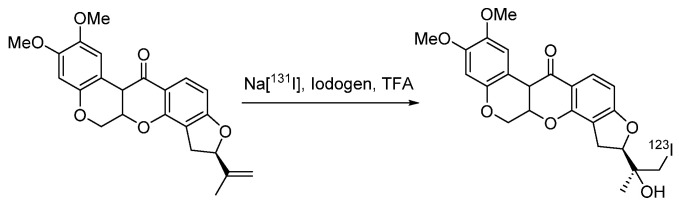
Synthesis of radiolabeled (2*R*)-2-(2-Hydroxy-1-iodopropan-2-yl)-8,9-dimethoxy-1,2,12,12a-tetrahydrochromeno [3,4-b]fluoro [2,3- h]chromen-6(6aH)-one [172].

A phase I clinical study of the radiopharmaceutical [^123^I]CMICE-013 was completed in 2012 to determine the safety, tolerability, pharmacokinetics, and imaging capabilities of the drug administered intravenously to healthy adult volunteers. As there is a need for alternatives to 99mTc-based perfusion radiopharmaceuticals for patients with cardiovascular disease, the [^123^I]CMICE-013 radiopharmaceutical has been investigated as an alternative indicator for measuring myocardial perfusion [172]. ^123^I-CMICE-013 was synthesized by radioactive labeling of rotenone in trifluoroacetic acid using iodogen as the oxidizing agent (Scheme 46). The reaction mixture was purified by reversed-phase HPLC to separate the product from Na^123^I, unlabeled rotenone, and other impurities. Analysis of the reaction mass using HPLC showed the presence of four radioactively labeled compounds, and the content of [^123^I]CMICE-013 was 74%.

#### 4.1.12. N-(2-(Diethylamino)ethyl)-6-iodoquinoxaline-2-carboxamide (ICF01012)

**Scheme 47:**
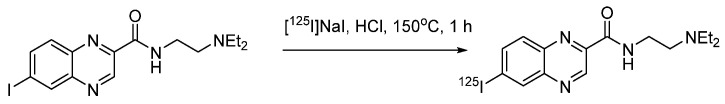
Radiolabeled N-(2-(diethylamino)ethyl)-6-iodoquinoxaline-2-carboxamide [175].

Benzamide-based radioligands that act on melanin were originally developed for melanoma imaging and later for therapeutic purposes in targeted radionuclide therapy. [^131^I]ICF01012 shows a very favorable in vivo pharmacokinetic profile for therapy. Preclinical studies in melanoma models have shown reduced tumor growth and improved survival. Based on these results, clinical trials are currently underway to determine the recommended dose of [^131^I]ICF01012 for the treatment of patients with pigmented metastatic melanoma [173]. In addition to labeling with iodine-131, N-(2-(diethylamino)ethyl)-6-iodoquinoxaline-2-carboxamide was also obtained for diagnostics based on iodine-123 and iodine-125 isotopes [174]. Radiolabeling of ICF01012 was carried out with [^123/125/131^I]NaI without the addition of a carrier using an isotope exchange reaction (Scheme 47). The resulting hydrochloride solution was evaporated to dryness, giving the product in an RCY of 77% and a radiochemical purity of 97% [175].

**Scheme 48:**
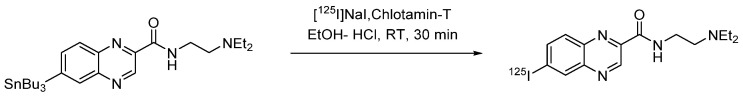
Synthesis of radiolabeled N-(2-(diethylamino)ethyl)-6-iodoquinoxaline-2-carboxamide [176].

A method for the introduction of iodine-125 or iodine-131 radioisotopes by the radioiododestannylation reaction of N-(2-diethylaminoethyl)-6-(tributylstannyl)quinoxaline-2-carboxamide was also developed (Scheme 48). [^131^I]ICF01012 was obtained with an RCY of 68% and radiochemical purity > 98%, while [^125^I]ICF01012 was obtained in an RCY of 52% and radiochemical purity > 99% [176]. It is also possible to use hydrogen peroxide as an oxidizing agent.

#### 4.1.13. Radioiodinated Drugs Based on Peptide Conjugates

**Scheme 49:**
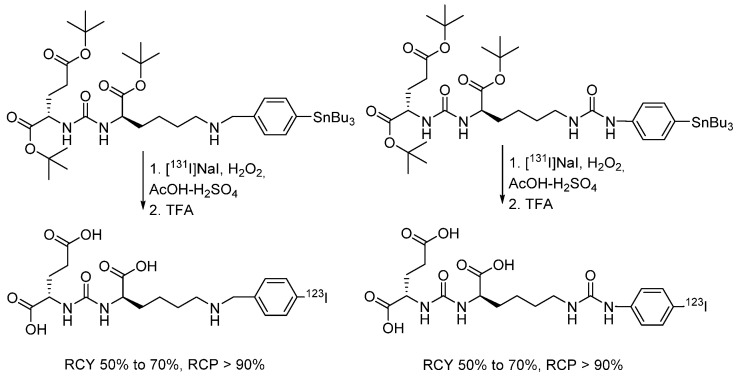
Synthesis of radiolabeled conjugates [^123^I]MIP-1072 and [^123^I]MIP-1095.

[^123^I]MIP-1072 and [^123^I]MIP-1095 conjugates are used for imaging for patients with metastatic prostate cancer. Phase I clinical trials for these compounds were completed in 2011. Radiolabeling procedure for the synthesis of [^123^I]MIP-1072 ([^123^I](S)-2-(3-((S)-1-carboxy-5-(4-iodobenzylamino)pentyl)ureido)pentanedioic acid) (Scheme 49) was carried out using [^123^I]NaI. The RCY ranged from 50% to 70%, and RCP was >90% [177]. [^123^I]MIP-1095 was obtained in a similar way. The resulting 123-I-MIP-1072 and 123-I-MIP-1095 are stable for 48 h at 40 °C.

### 4.2. Radioiodinated Drugs Based on Macromolecular Compounds

The database of clinical studies provides information on a significant number of antibodies that were labeled with radioisotopes of iodine, mainly iodine-131. In this section, we provide information only about such antibodies, which are currently at the stage of clinical research.

**^131^I-Omburtamab** (alternative names: ^131^I-8H9; 8H9-I-131; Burtomab I-131; Iodine-131 labeled monoclonal antibody 8H9; Monoclonal antibody-8H9-I-131). ^131^I-Omburtamab is a ^131^I-labeled mouse monoclonal antibody for the treatment of neuroblastomas in both adults and children. In March 2017, the European Medicines Agency designated ^131^I-8H9 as an orphan drug for the treatment of refractory leptomeningeal neuroblastoma metastases. In June 2017, ^131^I-Omburtamab received breakthrough therapy status for neuroblastoma (second-line therapy or higher, neonatal and pediatric metastatic disease) in the USA [178]. ^131^I-Omburtamab is also under clinical development for the treatment of CNS/leptomeningeal neuroblastoma metastases (BTD, ODD, and RPDD—phase II) and for the treatment of desmoplastic small round cell tumor (DSRCT—phase I). It is being investigated in parallel with the imaging agent ^124^I Omburtamab, which helps select patients with a potential positive response.

Labeling is carried out using an iodogen [179]. I*-Omburtamab with the activity up to 1295 MBq (35 mCi) was successfully prepared by the mixing of approximately 1 mg of unlabeled antibody and [^131^I]NaI or [^124^I]NaI, followed by purification on an anion exchange resin and microfiltration sterilization. The radiochemical purity of the obtained ^124^I-Omburtamaba was at least 90%, which was determined using thin-layer radiochromatography and the determination of the immunoreactive fraction using the analysis of live antigen-expressing cells. The finished single dose for a patient was 74 MBq (2.0 mCi) of ^124^I-Omburtamab containing 1.0 mg of a monoclonal antibody in 0.8–2.2 mL of a 3–5% HSA solution in phosphate buffer. The specific activity of the therapeutic antibody was 1295–1850 MBq/mg (35–50 mCi/mg).

**Iomab-AST** (alternative names: 131-I-labeled anti-CD45 antibody; 131-I-labeled BC8 antibody; ^131^I-BC8 antibody (anti-CD45); monoclonal antibody to CD45-BC8-I-131; monoclonal antibody to CD45-I-131; BC8; BC8-I-131; I-131-BC8; I-131-apamistamab; Iomab-ACT; and Iomab-B). ^131^I-Iomab-AST ([^131^I]BC8, Iomab-B™)) is a CD45 radioimmunotherapy drug in the treatment of cancer, particularly acute myeloid leukemia. Indications for use in acute myeloid leukemia, myelodysplastic syndrome, chronic myelomonocytic leukemia, and recurrent medulloblastoma were studied in the first clinical trials of ^131^I-BC8. ^131^I-Iomab-AST was successfully used as a myeloconditioning agent in more than 250 patients with incurable blood cancer. In phase I and II trials, ^131^I-Iomab-AST resulted in an effective cure for patients with no other treatment options left. The drug may also be effective in myelodysplastic syndrome, acute lymphoblastic leukemia, Hodgkin’s disease, and non-Hodgkin’s lymphoma. The current clinical trial includes elderly patients with relapsed and refractory acute myeloid leukemia who are being prepared for hematopoietic stem cell transplantation (often called bone marrow transplantation). The drug may enter the market by 2022–2023. Radiolabeling is carried out using chloramine-T [180,181,182].

## 5. Conclusions

Analyzing the synthetic methods used to obtain iodine-containing radiopharmaceuticals, the following main patterns can be noted:

- Electrophilic and nucleophilic substitution reactions, especially the former, are the predominant synthetic approaches used in radioiodination.
- Iododestantnylation is the best and most widely used method for electrophilic radioiodination of low-molecular-weight compounds, provided that the product can be purified from the unreacted tin-containing precursor.
- Fixing tin precursors to insoluble resins or using ionic liquids may overcome the difficulties of separation from toxic tin derivatives.
- Isotopic exchange of iodine-127 is the most commonly used method of nucleophilic radioiodination of low-molecular-weight compounds.
- Direct radioactive iodination of peptides and proteins is very easy, and it cannot be recommended as a method for the synthesis of radiopharmaceuticals, because the products are rapidly deiodinated in vivo.
- The problem of deiodination of proteins and peptides is generally solved by the use of prosthetic groups.

The main advantages of radioiodinated drugs are the optimal half-life of iodine isotopes ^123^I, ^124^I, ^125^I, and ^131^I; their selective accumulation in the tissues of the thyroid gland, stomach, and salivary glands; their attractive pharmacological and pharmacokinetic properties; their high efficiency of therapy with radioiodine-containing drugs in a number of diseases; and, as a rule, good tolerance by patients. The main obstacle to the wide use of radioactive iodine preparations is the limited possibility of synthesizing them under mild conditions and with acceptable chemical and RCYs. In this regard, it seems promising to develop convenient reproducible methods for the synthesis of the necessary precursors without the use of HPLC during purification.

Further development of metal-catalyzed radioiodination methods can be expected. There are prerequisites that, in the coming years, some of these methods, primarily azide–alkyne cycloaddition reactions, may find application in commercial synthesis of radiopharmaceuticals.

The development of molecular biology methods for incorporating various nonnatural amino acids into proteins will contribute to the creation of radioiodination methods with the possibility of introducing radioactive iodine as part of a prosthetic agent at a known position (far from the antigen-antibody binding site).

Finally, the availability of the latest hybrid imaging systems is another important direction in the development of the chemistry of radioiodinated compounds, regarding the creation of radioiodine-containing multimodal preparations for radionuclide imaging and therapy, as well as multimodal imaging.

## Figures and Tables

**Table 1:** Comparative characteristics of SPECT and PET techniques.

Characteristic	SPECT	PET
Equipment	γ-Camera	Tomograph
Radioisotopes	γ-Emitters	β+-emitters
Average procedure duration	30–40 min	10–20 min
Image reconstruction	Automatic	automatic
Spatial resolution	12–16 mm	4–6 mm
Sensitivity	Lower than that of PET	High
Multimodality	It is possible to combine SPECT-CT and SPECT-MRI	It is possible to combine PET-CT and PET-MRI
Availability	Wide	Less available than SPECT
Resulting image	2D or 3D reconstruction	3D
Quantification	Only semiquantitative assessment	Possible
Dose load on tissues	Lower than that of PET	Higher, but balanced by higher sensitivity

**Table 2:** Iodine isotopes used in nuclear medicine.

Radioisotope	^123^I	^124^I	^125^I	^131^I
Half-lifetime T_1/2_	13.22 h	4.18 days	59.39 days	8.02 days
Decay type	EC (100%)	EC (77%) β^+^ (23%)	EC (100%)	β^−^ (90%) EC (10%)
Energy of emitted particles *	γ (159 keV), 28.4 e^−^/decay (Auger electrons)	γ (603 keV) γ (511 keV upon annihilation of β^+^ with an electron) β^+^ (213.8 keV)	γ (35.5 keV), 19.5 e^−^/decay (Auger electrons)	γ (364 keV), β^−^ (606 keV)
Production **	Cyclotron: ^124^Xe(p, pn) ^123^Xe → ^123^I ^124^Xe(p, 2n) ^123^Cs→ ^123^Xe → ^123^I	Cyclotron: ^124^Te(p, n) → ^124^I	Nuclear reactor: ^124^Xe(n, γ) → ^125m^Xe → ^125^I; ^124^Xe (n, γ) → ^125g^Xe → ^125^I	Nuclear reactor: ^131^Te(n, γ) → ^131^I or ^235^U fission
Application	SPECT, radiotherapy	PET	Radiotherapy in vitro and in vivo experiments on small animals	Radiotherapy, SPECT
Ref.	[25]	[26]	[27]	[28]

* For the most probable decay pathway. ** The most common technique. β^+^—positron emission, EC—electron capture, β^−^—electron emission, PET—positron emission tomography, SPECT—single photon emission computed tomography.

## Data Availability

Not applicable.

## Abbreviations

A85380	5-Iodo-3-(2(S)-azetidinylmethoxy)pyridine
AGI-5198	N-Cyclohexyl-2-(N-(3-iodophenyl)-2-(2-methyl-1H-imidazol-1- yl)acetamido)-2-(o-tolyl)acetamide
BMIPP	β-Methyliodophenylpentadecanoic acid
β-CIT	2β-Carbomethoxy-3β-(4-iodophenyl)tropane
CLR-131	Iopofosin I-131, 12-[4-(^131^I)iodophenyl]dodecylphosphocholine
CMICE-013	(2R)-2-(2-Hydroxy-1-iodopropan-2-yl)-8,9-dimethoxy-1,2,12,12a- tetrahydrochromeno [3,4-b]fluoro [2,3-h] chromen-6(6aH)-one
CNS1261	N-(1-Naphthyl)-N’-(3-iodophenyl)-N-methylguanidine
(1-)-DABI	(4-isothiocyanatobenzylammonio)-undecahydro-closo-dodecaborate
FDA	Food and Drug Administration (Food and Drug Administration, USA)
FP-CIT	Ioflupane, N-ω-fluoropropyl-2β-carbomethoxy-3β-(4- iodophenyl)northropan
HAS	Human serum albumin
IAZA	1-(5-Iodo-5-deoxy-β-D-arabinofuranosyl)-2-nitroimidazole
IBOX	2-(4’-Dimethylaminophenyl)-6-iodobenzoxazole
IBM	N-(2-Aminoethyl)maleimide)
IBVM	Iodobenzovemicol
ICF01012	N-(2-(Diethylamino)ethyl)-6-iodoquinoxaline-2-carboxyamide ()
IMPY, TZDM	4-(6-Iodimidazo [1,2-a]pyridin-2-yl)-N,N-dimethylaniline
IMTO	Iodomethomidate
IMT	L-3-Iodine-α-methyltyrosine
IPA	para-I-Iodo-L-Phenylalanine
IPBM	2-((2-Iodophenyloxy)(phenyl)methyl)-2-methylmorpholine
IPM	1-(3-Iodophenyl)maleimide
IPPA	para-Iodophenylpentadecanoic acid
mAb	Monoclonal antibodies
MAPi	4-(4-Fluoro-3-(4-(3-iodobenzoyl)piperazine-1-carbonyl)benzyl) phthalazin-1(2H)-one
MIBG, MIBG	meta-Iodobenzylguanidine
MIP-1072	(S)-2-(3-((S)-1-carboxy-5-(4-iodobenzylamino)pentyl)ureido) pentanedioic acid
MNI-187	4-(5-Iodo-2H-benzo[d][1,2,3]triazol-2-yl)-N,N-dimethylaniline
MZINT	2β-Carbomethoxy-3β-(4′-((Z)-2-iodoethenyl)phenyl)northropan
NBS	N-Bromosuccinimide
NCS	N-Chlorosuccinimide
NCTFS	N-Chloro-tetra-fluorosuccinimide
PET	Positron emission tomography
PIB	N-Succinimidyl-4-iodobenzoate
RCC	Radiochemical Conversion
RCP	Radiochemical purity
RCY	Radiochemical yield
SIB	N-Succinimidyl-3-iodobenzoate
SIPC	N-Succinimidyl-5-iodine-3-pyridinecarboxylate
SPECT	Single photon emission tomography
TCP	2,3,5,6-Tetrafluorophenyl-3-(nidocarboranyl)propionate
TFIB	Tetrafluorophenyl-4-fluoro-3-iodobenzoate.
